# Metabolomics workbench: data-driven classification and correlation analysis between metabolite classes, biomarkers, and drugs

**DOI:** 10.1093/bib/bbag576

**Published:** 2026-10-19

**Authors:** Mikhail M Khandokhin, Aleksandr Sokolov, Nikolay Oskolkov, Robert Fredriksson, Olga E Titova, Helgi B Schiöth

**Affiliations:** Department of Pharmaceutical Biosciences; Pharmacology, Uppsala University, Husargatan 3, SE-751 24 Uppsala, Sweden; Department of Surgical Sciences, Functional Pharmacology and Neuroscience, Uppsala University, Husargatan 3, SE-751 24 Uppsala, Sweden; Metabolic Research Group, National Institute of Research and Innovation, Aizkraukles iela 21, LV-1006 Riga, Latvia; Department of Pharmaceutical Biosciences; Pharmacology, Uppsala University, Husargatan 3, SE-751 24 Uppsala, Sweden; Department of Medical Sciences, Molecular Epidemiology, Uppsala University, Dag Hammarskjölds väg 14B, SE-751 85 Uppsala, Sweden; Department of Clinical Science and Education, Södersjukhuset, Karolinska Institutet, Sjukhusbacken 10, SE-118 83 Stockholm, Sweden; Department of Surgical Sciences, Functional Pharmacology and Neuroscience, Uppsala University, Husargatan 3, SE-751 24 Uppsala, Sweden; Metabolic Research Group, National Institute of Research and Innovation, Aizkraukles iela 21, LV-1006 Riga, Latvia

**Keywords:** metabolomics, meta-analysis, human, blood, biomarkers, drugs

## Abstract

Public repositories now accumulate an unprecedented amount of metabolomics data. Despite the opportunities for large-scale meta-analyses opened by these repositories, the deposited data rarely becomes systematically re-analyzed. We conducted a systematic metadata-based re-analysis of all human blood metabolomics studies in the Metabolomics Workbench performed using Orbitrap or QTOF mass spectrometry, encompassing 44 165 samples. After harmonizing metabolites with PubChem identifiers and embedding them using SMILES representations, we generated a map of 6752 blood metabolites forming 25 structural clusters, and subsequently performed correlation analysis between these clusters, biomarkers, and drugs. Analysis of 337 120 metabolite pairs detected in at least 10 studies showed that structurally related clusters display correlated abundances (ρ up to 0.6). Correlation patterns among established clinical biomarkers revealed candidate metabolic correlates, several with ρ > 0.7. We also developed open-source software enabling similar analyses for any metabolite across the Workbench data explored. Additionally, curating drug-containing datasets enabled meta-analyses for compounds such as acetaminophen, metoprolol, and atenolol, identifying both known and novel metabolic signatures. Besides the biological insights, we have found that Orbitrap and QTOF tend to detect different metabolites, but they give quantitatively coherent results on overlapping compounds. Overall, the present study provides the methodology and software to perform large-scale mining of public metabolomics data enabling its re-analysis to obtain novel biomedical insights.

## Introduction

Metabolomics has become central to systems biomedicine and drug discovery because it provides a functional readout that complements genomics, transcriptomics, and proteomics offering comprehensive measurement of small molecules in cells, tissues, and several biofluids [1]. Recent advances in mass spectrometry (MS) / nuclear magnetic resonance (NMR) workflows and analytics have accelerated both discovery and targeted analyses, from structure-to-function “reverse metabolomics” strategies to unified liquid chromatography–mass spectrometry (LC–MS) pipelines that improve transparency, quality control (QC), and reproducibility [2, 3]. In parallel, open repositories have matured: the National Metabolomics Data Repository (NMDR; “Metabolomics Workbench”) now aggregates numerous researcher-deposited datasets alongside other resources such as EMBL-EBI’s MetaboLights and DDBJ’s MetaboBank [4, 5]. These resources are supported by validation and metadata services along with pan-repository reanalysis frameworks that enable robust cross-study mining and benchmarking [6, 7].

The Metabolomics Workbench now houses thousands of studies covering human cohorts, model organisms, plants, and microbes (accessed 2025). While these datasets include a wide spectrum of experimental designs, analytical platforms, and biological contexts, only a fraction have been reanalyzed beyond their original publications. This creates a substantial, largely untapped opportunity for integrative research: large-scale meta-analyses, comparative studies across species, and the discovery of novel links between metabolite classes, disease biomarkers, and drug response. The combination of raw data, curated metadata, and direct connections to other resources such as PubChem and Gene Expression Omnibus position Metabolomics Workbench as a uniquely powerful foundation for secondary analyses that could reveal patterns and hypotheses not apparent in individual studies [6–8].

In this study, we mined the Metabolomics Workbench to map its data content, analyze the principled classification of chemical groups, and examine correlations among metabolite classes. We also created an easy-to-use tool to analyze correlations between available individual metabolites, compared the Orbitrap and QTOF platforms, and explored translational links to biomarkers and drugs.

## Methods

Please refer to Supplementary Information for the Extended Methods section.

### Ethics

This work has been conducted using data from primary human studies publicly deposited at Metabolomics Workbench. These studies were approved by corresponding regional ethical committees as indicated in their primary publications. Data analysis and processing have been approved by Swedish Ethical Review Authority (EPM).

### Data source

All metabolomics datasets used in the present study have been retrieved from the National Metabolomics Data Repository (NMDR, “Metabolomics Workbench”) on June 7, 2025. The datasets could be accessed on https://www.metabolomicsworkbench.org.

### Code availability

All code related to this work as well as the tool to identify metabolic correlates could be found at GitHub repository https://github.com/FunctPharm-NeuroUU/metabolomics_workbench_meta-analysis

### Study selection

The metadata of studies from Metabolomics Workbench database was retrieved on June 7, 2025 using the REST service. Studies were filtered with the parameters: species—human, sample type—blood, instrument type—Orbitrap or QTOF (Fig. 1, Supplementary Fig. 1). This subset of 182 studies was used subsequently in all further processing steps. Giving the heterogeneity of the included studies and incomplete reporting of the quality control samples in public repositories, we did not measure data quality and filter the studies based on it. The robustness of the analysis was ensured by the within-study analyses, non-parametric statistics and large-scale meta-analytic approach.

**Figure 1:**
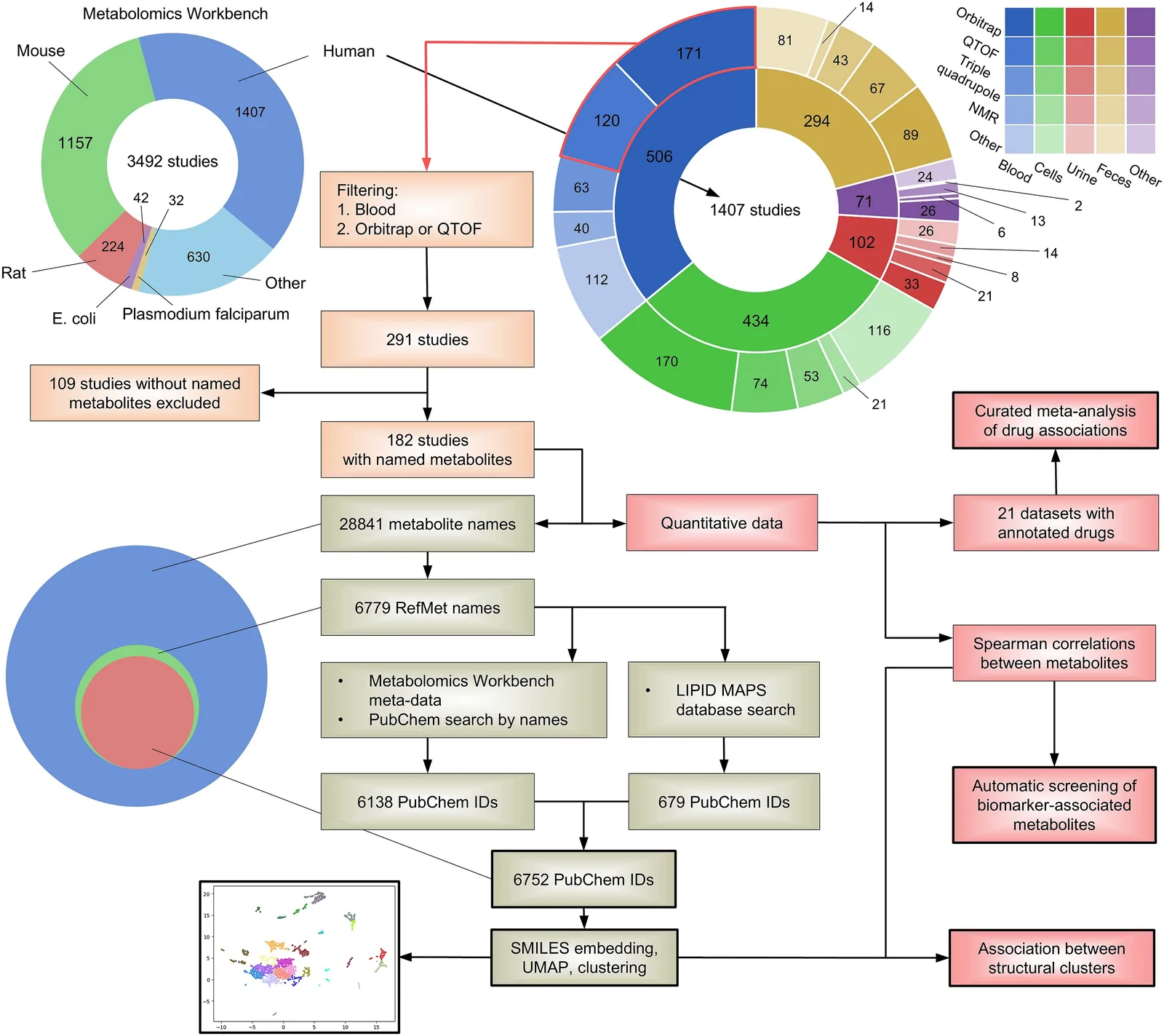
Metabolomics workbench repository content and pipeline of analyses performed. A pie chart at the top left corner visualizes the distribution of studies on metabolomics workbench by species, while a pie chart at the top right corner visualizes the distribution of human studies by sample type and analysis method. The color of text boxes in the flow chart represents a stage of analysis: orange—filtering, green—structural analysis based on SMILES, and red—quantitative analyses.

### Data retrieval

Metabolomics Workbench has an internal unified metabolite name system ascribing special RefMet names to almost all metabolites in a study, which helps to prevent the issue of a single metabolite being named by different synonyms in different studies. However, as one of our aims was to perform structural embedding and clustering based on SMILES (Simplified Molecular Input Line Entry System, line notation for chemical structure), which is not reported in many cases in Metabolomics Workbench metadata, we decided to retrieve SMILES from the PubChem database [9]. Metabolite names and RefMet names (internal unified names given by Metabolomics Workbench database to address the challenge of synonyms) from the studies selected previously have been used to retrieve PubChem IDs. Briefly, PubChem IDs have been retrieved from Metabolomics Workbench metadata, if present, and from PubChem and LIPID MAPS [10] databases using search by names. The resulting PubChem IDs were used to retrieve SMILES from the PubChem database. Except for curated drug intake association analysis, we did not normalize the reported metabolite abundance data, because of the large number of included studies and considerable difficulties that would be associated with this approach due to heterogeneity. Instead, the comparability was ensured by rank-based statistics and meta-analytic aggregation on a level of study-wise non-parametric statistics (Spearman’s correlation coefficients).

### Metabolite structural embedding by SMILES

Structural embedding of metabolites was performed using the ChemBERTa-77M-MTR [11] model. The 6752 SMILES strings associated with previously retrieved PubChem CIDs were embedded and the resulting 600-dimensional vectors for each SMILES were subjected to dimension reduction using Principal Component Analysis (PCA) and Uniform Manifold Approximation and Projection (UMAP). PCA and UMAP have been chosen as common examples of linear and non-linear methods for dimension reduction, respectively.

### Metabolite structural clustering

We aimed to perform structural clustering of metabolites based on SMILES embeddings and compared several clustering methods: k-means on PCA-reduced embeddings, bisecting k-means on PCA-reduced embeddings, agglomerative (hierarchical) clustering on PCA-reduced embeddings, and k-means on UMAP-reduced embeddings.

To evaluate clustering performance, we retrieved chemical class annotations using ClassyFire web service [12] (Supplementary Fig. 3). As better clustering would generate more homogenous clusters in terms of the present main metabolite classes, we quantified cluster homogeneity using the Gini impurity metric (1)


(1) \begin{eqnarray*} Gini\ impurity=1-\varSigma{p}_i^2 \end{eqnarray*}

where *p_i_* is the frequency of the *i^th^* main metabolite class within a cluster. Gini impurity was calculated for each cluster and then averaged to produce an overall clustering quality metric.

Despite concerns about using UMAP-reduced data for clustering [13], the evaluation demonstrated that k-means clustering on UMAP-reduced embeddings in our particular case produced the most homogeneous clusters (mean Gini impurity = 0.737) compared with all PCA-based methods (mean Gini impurity = 0.876, 0.894, 0.865; Supplementary Fig. S4). Based on these results, we selected k-means clustering on UMAP-reduced data for all subsequent analyses.

### Correlation analysis between chemical classes

After clustering metabolites into 25 chemical clusters, we aimed to estimate the correlations between these clusters. Given the heterogeneity of the included studies, including differences in cohorts, instrumentation, and measurement units, we decided to use the nonparametric approach using Spearman’s rank correlation coefficient. Correlations between clusters were derived by aggregating all available metabolite–metabolite correlations for a cluster pair across individual studies.

To estimate the correlation between two chemical clusters (cluster₁, cluster₂), we refined all metabolite pairs (metabolite₁ ∈ cluster₁, metabolite₂ ∈ cluster₂) that were reported in at least 10 studies. For each metabolite pair, individual Spearman’s correlation coefficients were aggregated by calculating a weighted mean of primary Spearman’s correlations per study (with sample sizes in studies being the weights) and *P*-values were aggregated by taking a median *P*-value of individual primary *P*-values (metabolite-level aggregation):


(2) \begin{eqnarray*} {\rho}_{metabolite\ pair}=\frac{\sum_{i=1}^n{\rho}_{study\ i}\times{sample\ size}_i}{\sum_{i=1}^n{sample\ size}_i} \end{eqnarray*}

(3) \begin{eqnarray*} p-{value}_{metabolite\ pair}= median\left(p-{value}_{individual\ studies}\right) \end{eqnarray*}

To calculate the final estimate of correlation between chemical clusters, aggregated Spearman’s correlation coefficients were aggregated for the second time by calculating a weighted mean (with sums of sample sizes in studies being the weights) and *P*-values were aggregated by taking a median (cluster-level aggregation):


(4) \begin{eqnarray*} {\rho}_{cluster\ pair}=\frac{\sum_{i=1}^n{\rho}_{metabolite\ pair\ i}\times sum\ {sample\ size}_i}{\sum_{i=1}^n sum\ {sample\ size}_i} \end{eqnarray*}

(5) \begin{eqnarray*} p-{value}_{cluster\ pair}= median\left(p-{value}_{metabolite\ pair}\right) \end{eqnarray*}

The estimates of intracluster correlation were also calculated using the same approach but restricting both metabolites to the same cluster.

As the method of aggregation may impact subsequent analysis and given that there is no universally accepted way to aggregate Spearman’s correlation coefficients, we performed sensitivity analysis of the aggregation method by comparing results obtained using weighted mean, unweighted mean, and median aggregation of Spearman’s coefficients (Supplementary Fig. 6). The results were robust, showing no significant differences across aggregation methods.

### Automated screening for metabolite associations

To enable access to Metabolomics Workbench data and facilitate rapid hypothesis testing of metabolite associations and the identification of potential metabolic correlates or structurally similar compounds, we developed a software tool accessible through GitHub (see Code availability section). The software utilizes Spearman’s correlation data for all possible metabolite pairs present in the studies analyzed in this work. Details about installation and usage may be found in GitHub repository.

This automated screening tool in the second mode was used to generate the results presented in Table 1, which lists the metabolic correlates for clinically measured biomarkers.

**Table 1:** Metabolic proxies for clinically measured biomarkers found by automated correlation analysis.

Biomarker	Functional type	Association type	Metabolite associated	Weighted Spearman’s correlation coefficient	Median *P*-value	Number of studies
Homocysteine	Cardiovascular	Positive (top 1)	Linoleoyl-CoA	0.870917	8.29E-05	5
Homocysteine	Cardiovascular	Positive (top 2)	N-Acetylornithine	0.457872	0.009586255	10
Homocysteine	Cardiovascular	Positive (top 3)	CAR 4:0;3OH	0.316303	0.035674234	14
Homocysteine	Cardiovascular	Negative (top 1)	Acetaminophen	−0.039978	0.010717508	7
Homocysteine	Cardiovascular	Negative (top 2)	CAR 5:0	0.004613	0.003998103	7
Homocysteine	Cardiovascular	Negative (top 3)	Hydroxykynurenine	0.019823	0.014908742	5
Trimethylamine N-oxide	Cardiovascular	Positive (top 1)	6-Hydroxyindole sulfate	0.466503	4.43E-10	18
Trimethylamine N-oxide	Cardiovascular	Positive (top 2)	Argininosuccinic acid	0.460067	0.00197109	6
Trimethylamine N-oxide	Cardiovascular	Positive (top 3)	alpha-N-Phenylacetylglutamine	0.455742	5.66E-12	37
Trimethylamine N-oxide	Cardiovascular	Negative (top 1)	1,5-Anhydrosorbitol	−0.124372	0.00680169	27
Trimethylamine N-oxide	Cardiovascular	Negative (top 2)	Cryptoxanthin	−0.105198	0.0231857	19
Trimethylamine N-oxide	Cardiovascular	Negative (top 3)	HexCer 18:2;O2/22:0	−0.099082	0.000641698	9
Hypoxantine	Cardiovascular	Positive (top 1)	Fructose 6-phosphate	0.635732	0.00172042	6
Hypoxantine	Cardiovascular	Positive (top 2)	Deoxyinosine	0.533034	1.02E-39	5
Hypoxantine	Cardiovascular	Positive (top 3)	Inosine	0.487402	9.21E-06	83
Hypoxantine	Cardiovascular	Negative (top 1)	LPE 18:3	−0.230783	0.0497413	5
Hypoxantine	Cardiovascular	Negative (top 2)	1-Pyrroline-5-carboxylic acid	−0.206094	0.0238686	21
Hypoxantine	Cardiovascular	Negative (top 3)	bis(2-Ethylhexyl)phthalic acid	−0.156748	0.0186507	5
Pseudouridine	Cancer	Positive (top 1)	5,6-Dihydrouridine	0.825147	5.68E-72	20
Pseudouridine	Cancer	Positive (top 2)	3-(3-amino-3-carboxypropyl)uridine	0.732898	4.70E-80	10
Pseudouridine	Cancer	Positive (top 3)	C-Glycosyltryptophan	0.727272	4.00E-36	24
Pseudouridine	Cancer	Negative (top 1)	PC 34:4	−0.162772	0.0092064	5
Pseudouridine	Cancer	Negative (top 2)	LPE 20:0	−0.136471	0.0249739	6
Pseudouridine	Cancer	Negative (top 3)	LPC 14:0	−0.126646	0.00825218	10
5-Hydroxyindoleacetic acid	Cancer	Positive (top 1)	Aniline	0.48106	5.00E-42	8
5-Hydroxyindoleacetic acid	Cancer	Positive (top 2)	Kynurenine	0.441888	9.23E-05	54
5-Hydroxyindoleacetic acid	Cancer	Positive (top 3)	Succinyladenosine	0.401541	0.0167429	8
5-Hydroxyindoleacetic acid	Cancer	Negative (top 1)	Folic acid	−0.178035	0.0446093	5
5-Hydroxyindoleacetic acid	Cancer	Negative (top 2)	5-HETE	−0.119289	0.0444509	5
5-Hydroxyindoleacetic acid	Cancer	Negative (top 3)	LPI 20:4/0:0	−0.0916996	0.013187	5
2-hydroxyglutarate	Cancer	Positive (top 1)	Gluconolactone	0.61941	9.29E-08	5
2-hydroxyglutarate	Cancer	Positive (top 2)	8-Hydroxyoctanoic acid	0.549664	6.51E-42	6
2-hydroxyglutarate	Cancer	Positive (top 3)	Dehydroascorbic acid	0.504273	2.90E-05	15
2-hydroxyglutarate	Cancer	Negative (top 1)	3,5-Dichloro-2,6-dihydroxybenzoic acid	−0.107514	0.0163291	8
2-hydroxyglutarate	Cancer	Negative (top 2)	1H-Indole-7-acetic acid	−0.0949563	0.0365208	6
2-hydroxyglutarate	Cancer	Negative (top 3)	SM 18:2;O2/18:1	−0.0603303	0.0112453	12
Sarcosine	Cancer	Positive (top 1)	Iminodiacetate	0.519134	3.77E-23	9
Sarcosine	Cancer	Positive (top 2)	Alanine	0.372051	0.0118748	37
Sarcosine	Cancer	Positive (top 3)	beta-Alanine	0.366863	0.0319335	29
Sarcosine	Cancer	Negative (top 1)	ADP	−0.300703	2.27E-06	5
Sarcosine	Cancer	Negative (top 2)	Palmitoylcholine	−0.177133	0.022071	18
Sarcosine	Cancer	Negative (top 3)	Oleoylcholine	−0.176471	0.0362175	18
Creatinine	Renal disease	Positive (top 1)	N,N,N-Trimethyl-alanylproline betaine	0.705977	3.03E-45	15
Creatinine	Renal disease	Positive (top 2)	Methylguanidine	0.655547	1.44E-13	15
Creatinine	Renal disease	Positive (top 3)	Leu-Pro	0.619319	0.00133427	8
Creatinine	Renal disease	Negative (top 1)	25-Hydroxyvitamin D3	−0.326975	2.99E-05	5
Creatinine	Renal disease	Negative (top 2)	PC 40:10	−0.263834	0.00494891	5
Creatinine	Renal disease	Negative (top 3)	CE 18:3	−0.154764	0.0187923	9

### Study screening for drug intake association analysis

We automatically screened the initial set of human blood Orbitrap- or QTOF-based studies for the presence of drugs or drug metabolites among the annotated compounds and found 79 drugs present in at least five studies and a total of 77 studies containing them.

The resulting 77 studies were screened manually, which allowed us to collect the information about the actual number of subjects (in contrast to the automatically accessible number of samples that may be technical replicates or timepoints related to the single subject), data processing and normalization procedures, and subject metadata (e.g. age, sex, and BMI). As a result of the screening, 24 studies were included in the analysis (Supplementary Table 1).

### Meta-analysis and mega-analysis of metabolites associated with drug intake

To harmonize the studies for meta-analysis and mega-analysis and ensure that all samples included in the analysis are actual biological replicates from independent subjects, several preprocessing stages were performed: technical replicates were averaged and timepoints were excluded except for baseline, median-batch normalization was performed if the dataset contained raw data (in all other included studies median-batch normalized data has been deposited on Metabolomics Workbench), missing values was imputed by a half minimum, and log-transformation was performed (dataset_preprocessing_1.R).

Datasets containing drug data were processed separately (dataset_preprocessing_2.R, dataset_preprocessing_3.R). All metabolite forms of a single drug, as well as all technical replicates, were averaged to yield a single estimate of drug usage. As most of the subjects with the detected drug concentration (which, for some datasets and drugs, were detected in all samples) were actual non-takers, we set a task to create a threshold to classify subjects as drug takers and non-takers. We selected a value twice the dataset median as a suitable threshold based on the examination of multiple cases of its performance by visual inspection of distribution patterns (Supplementary Fig. 8). The cases were coherent with the intuition of concentration of drug non-takers forming a peak in low values, and few actual drug takers being in the high value zone. Finally, we merged processed datasets of binary drug intake data and non-drug metabolite concentrations, and added confounders (sex, age, and BMI, if present) from metadata (dataset_preprocessing_4.R). We decided to perform the analysis for drugs present in at least five cohorts, which refined 10 drugs: acetaminophen, metoprolol, atenolol, hydrochlorothiazide, naproxen, metformin, ibuprofen, ranitidine, gabapentin, and diphenhydramine.

To aggregate the data between studies, we decided to test two approaches: random-effects meta-analysis and mega-analysis. For meta-analysis, ANCOVA was used to calculate the association of drug intake and metabolite concentration for each metabolite in each study. Age, sex and BMI were included as confounders in the model if they were present in the dataset metadata. LogFC and *P*-values for each metabolite and for each study were meta-analysed by random-effect model using Sidik-Jonkman estimator [14]. MS instrument type was included as a moderator if there were at least three studies with each instrument type. In parallel, mega-analysis was conducted in which subjects from all studies for a particular metabolite were combined in one ANCOVA model, and study was included in a model as a confounder.

### Functional annotation by MetaboAnalyst

Using the nominally significant (uncorrected *P* < .05) differential metabolites found by meta-analysis, functional annotation was performed in MetaboAnalyst 6.0 web-service [15].

## Results

### Metabolomics workbench repository data enables large-scale automated meta-analyses

As of June 7, 2025, the Metabolomics Workbench repository contained ~3500 publicly available metabolomics studies. By comparison, other major repositories: EMBL-EBI’s MetaboLights [4], DDBJ’s MetaboBank [5], and GNPS/MassIVE [16]—hosted around 1800, 2000, and 1200 studies, respectively, on the same date. This establishes the Metabolomics Workbench as the largest public repository of metabolomics studies to date.

To maximize comparability across datasets, we restricted our analysis to blood samples, the most common biofluid, and further refined the dataset to studies performed using Orbitrap or QTOF instruments. This approach reduces heterogeneity while preserving the ability to systematically compare these two widely used MS platforms. After applying an additional filter to include only studies with annotated metabolites, 182 datasets remained, encompassing 44 165 samples and 22 841 metabolite names prior to synonym matching (Fig. 1). The included datasets mainly utilized liquid chromatography (LC–MS) for metabolite separation (172 datasets), the other options were gas chromatography (GC–MS, five datasets) and both LC–MS and GC–MS (five datasets). Reversed phase (148 datasets) and Hydrophilic interaction chromatography (HILIC, 54 datasets) were mostly used. Orbitrap and QTOF instruments are almost exclusively represented by Thermo Fisher Scientific and Agilent Technologies products, respectively.

The primary objective of this study was to systematically characterize the human blood metabolome by structurally clustering detected metabolites, assessing global associations between major structural clusters, and investigating specific metabolome associations using clinically relevant examples such as biomarkers and drugs. In addition, we sought to compare the two most frequently used analytical modalities in the Metabolomics Workbench, Orbitrap and QTOF MS, in terms of metabolome coverage and quantification. The overall workflow consisted of two major components: structural data processing for metabolite clustering (Fig. 1, green) and quantitative analyses (Fig. 1, red).

Through the automated name-matching procedure (see Methods “Data retrieval” section for details), we obtained 6752 PubChem CIDs, covering the majority of metabolites reported across studies based on the total of 6779 unique RefMet names. Most PubChem CIDs appeared in only a small number of studies (Supplementary Fig. 1d). Nevertheless, 1351 CIDs were represented in at least 10 studies—already exceeding the number of metabolites reported in almost all individual datasets. Although our strict inclusion criteria reduced the number of studies compared with the full Metabolomics Workbench content, the analysis demonstrates a reproducible framework and accompanying software for large-scale, automated meta-analysis. This methodology is broadly applicable and can be extended to other datasets within the Metabolomics Workbench.

### The number of detected metabolites is determined primarily by the variety of technical settings within a study

Metabolomics, like other systems biology approaches, aims to capture and analyze the widest possible range of elements, i.e. metabolites. Large-scale comparative meta-analyses provide an opportunity to identify the factors that determine how many metabolites can be annotated in a given study. To explore this, we focused on the refined set of Orbitrap- and QTOF-based human blood studies from the Metabolomics Workbench.

We found statistically significant correlation between sample size and metabolite count using Spearman’s correlation (ρ = 0.25, *P* = .0008; Fig. 2a), although the effect size was small. These results suggest that sample size has little influence on the number of metabolites detected in human blood metabolomics studies.

**Figure 2:**
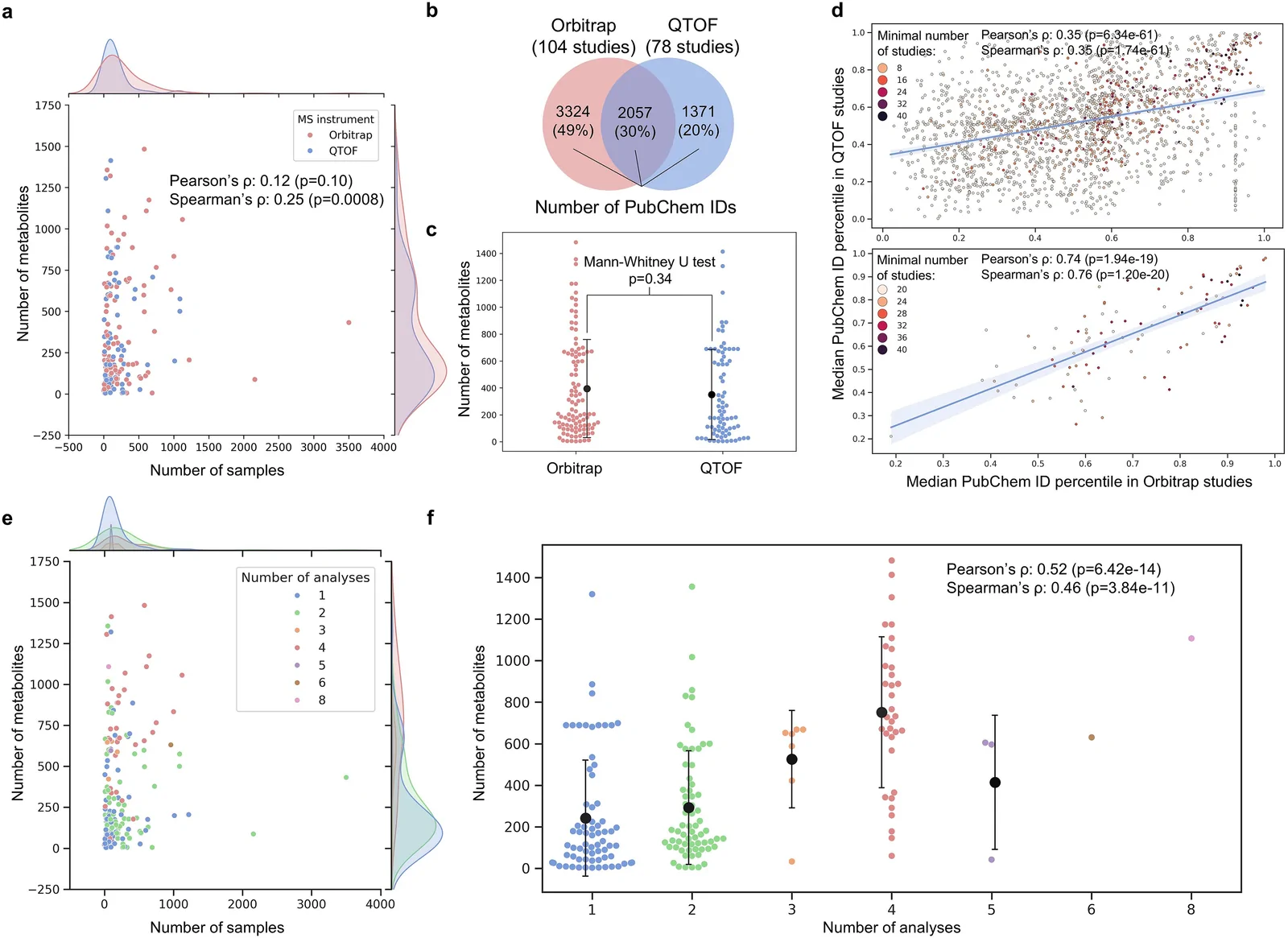
Technical characteristics of studies defining the coverage and quantification of metabolites. (a) Jointplot showing the relationship between the number of samples and the number of detected metabolites in a study. Correlation coefficients and their *P*-value for the relationship are given. Grouping is performed based on the MS type: Orbitrap or QTOF. (b) Euler diagram showing the number of PubChem CIDs detected only in Orbitrap studies (red zone), only in QTOF studies (blue zone), or in both types of studies (intersection). (c) Swarm plot showing the distribution of the number of detected metabolites in Orbitrap and QTOF studies. (d) Scatter plots showing the association between the median percentile of the metabolite concentration among all detected metabolites in Orbitrap and QTOF studies. (e) Joint plot showing the relationship between the number of samples and the number of detected metabolites in a study. Correlation coefficients and their *P*-value for the relationship are given. Grouping is performed based on the number of separate analyses with different processing. (f) Swarm plot showing the distribution of the number of detected metabolites in groups of studies by the number of separate analyses with different processing.

We next compared Orbitrap and QTOF MS modalities in terms of metabolome coverage and quantification. Among 104 Orbitrap-based studies and 78 QTOF-based studies, 3324 PubChem CIDs (49%) and 1371 PubChem CIDs (20%), respectively, were uniquely detected by one modality, while 2057 PubChem CIDs (30%) were shared between both (Fig. 2b). To test whether the low number of shared metabolites could be explained by stochastic reasons, given that many metabolites are detected only in a few studies, we performed the permutation test by mixing Orbitrap and QTOF labels between studies and calculating the simulated number of shared metabolites for 10,000 iterations. The observed number of shared metabolites is significantly lower than expected by chance (mean = 2959, *P* = .0). The total number of metabolites detected per study did not differ significantly between Orbitrap and QTOF (Mann–Whitney U test, *P* = .34; Fig. 2c). Thus, although the two instruments detect a similar number of metabolites, studies utilizing them cover partially different metabolites. Importantly, for metabolites detected by both modalities, reported concentrations (expressed as the median percentile across studies) were correlated (Pearson’s r = 0.35, *P* = 6.34 × 10^−61^; Spearman’s ρ = 0.35, *P* = 1.74 × 10^−61^). This correlation strengthened when restricting the analysis to metabolites reported in at least 20 studies per modality (Pearson’s r = 0.74, *P* = 1.94 × 10^−19^; Spearman’s ρ = 0.76, *P* = 1.20 × 10^−20^; Fig. 2d, Supplementary Fig. 2). These findings indicate that while Orbitrap and QTOF studies cover partially different metabolites, their quantification of overlapping metabolites is consistent.

Finally, we investigated whether the combination of several technical settings within a study contributes to metabolite detection. Most Metabolomics Workbench studies report multiple analyses per sample, differing in chromatography type (e.g. HILIC, reverse-phase liquid chromatography) and ion mode (positive or negative). We observed an expected trend that the number of distinct technical settings combined in a study strongly correlated with the number of detected metabolites (Pearson’s r = 0.52, *P* = 6.42 × 10^−14^; Spearman’s ρ = 0.46, *P* = 3.84 × 10^−11^; Fig. 2e and f). Taken together, these results suggest that the most effective way to broaden metabolome coverage for meta-analysis is to include studies with diverse technical settings including instrument type, chromatography, and ion mode, although the balance should be found to limit the heterogeneity.

### Structural clustering and correlation analysis of 6752 blood metabolites reveal that structurally related compounds exhibit correlated abundances

To cluster metabolites from Orbitrap- and QTOF-based human blood studies on the Metabolomics Workbench, we used embeddings of their SMILES representations by the ChemBERTa model. SMILES strings were retrieved from the PubChem database for 6752 PubChem CIDs identified previously. Because ChemBERTa-generated output is high-dimensional, we applied dimensionality reduction using both PCA and UMAP, followed by several clustering algorithms (Fig. 3b). Although UMAP-based clustering has been debated [13], several studies have shown that it can enhance performance in certain datasets [17, 18]. In our particular case, k-means clustering on UMAP-reduced embeddings yielded the most homogeneous clusters, as measured by the Gini impurity metric with PubChem chemical classes as a validation metric (Supplementary Figs 3 and 4). Therefore, we selected this approach for downstream analyses.

**Figure 3:**
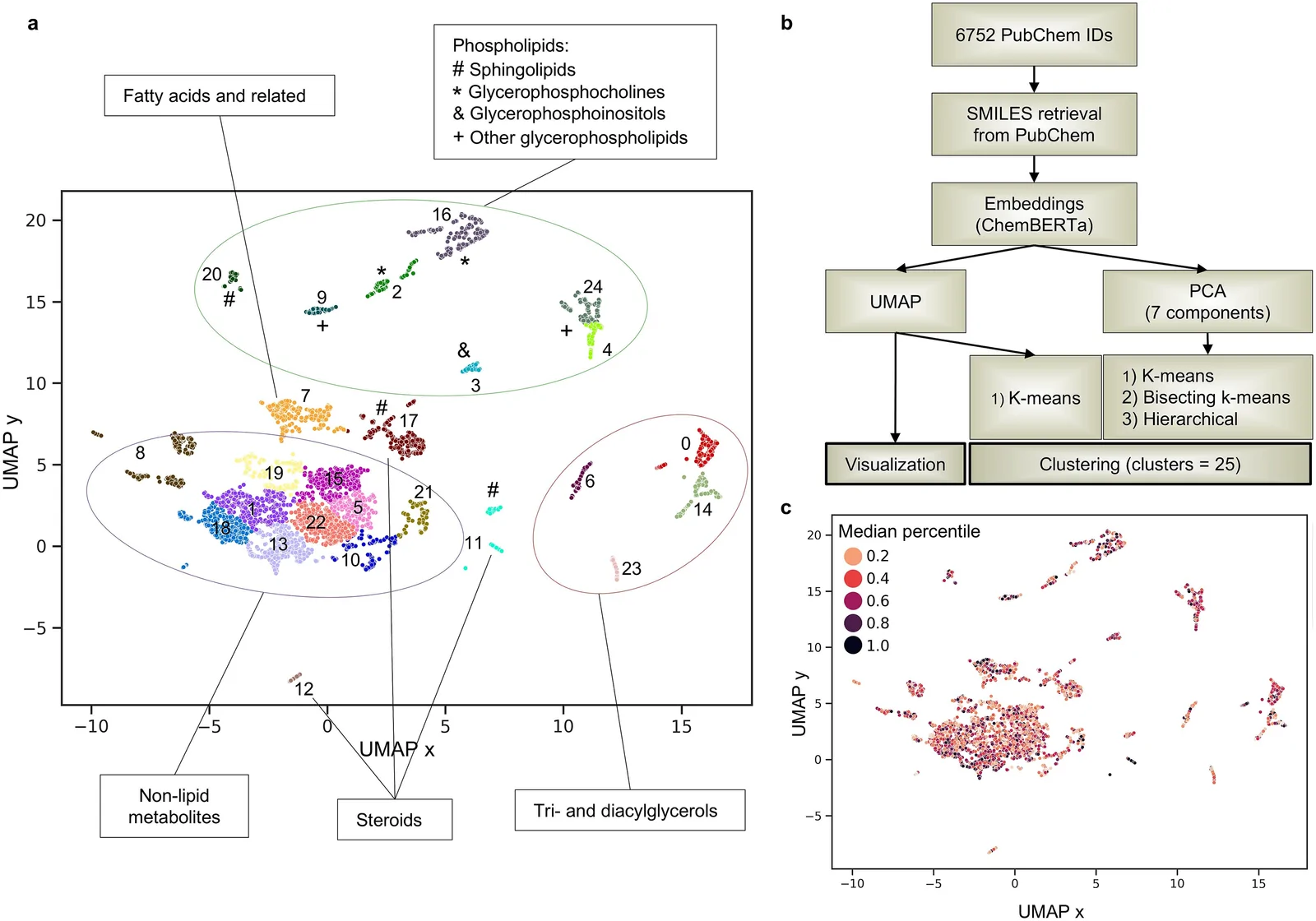
Structural clustering of 6752 metabolites in blood revealing present chemical classes. (a) UMAP generated on the SMILES embeddings by the ChemBERTa model colored by the cluster number. (b) Detailed pipeline of the structural analysis. (c) UMAP generated on the SMILES embeddings by the ChemBERTa model colored by the median percentile of the metabolite concentration among all detected metabolites.

Structural clustering revealed that the human blood metabolome encompasses a broad diversity of chemical classes (Fig. 3a, Supplementary Fig. 5). Abundant and less represented metabolites appeared to be distributed relatively evenly across clusters (Fig. 3c). Overall, the numbers of lipid and non-lipid metabolites in blood detected by Orbitrap- and QTOF-based studies are balanced: 2805 lipids (mainly in clusters 0, 2–4, 6–7, partly 8, 9, 11–12, 14, 16–17, 20, 23–24) and 3947 non-lipid metabolites (mainly in clusters 1, 5, partly 8, 10, 13, 15, 18–19, 21–22). Lipid clusters included acylglycerols ranging from triacylglycerols (clusters 0, 14, 23) to diacylglycerols (cluster 6); sphingolipids (clusters 20, partly 11, 17); glycerophospholipids, including glycerophosphocholines (clusters 2, 16), glycerophosphoethanolamines (partly clusters 4, 9, 24), glycerophosphoserines (partly clusters 4, 9, 24), glycerophosphoinositols (cluster 3), cardiolipins (partly cluster 0); steroids (cluster 12, partially clusters 11, 17); acylcarnitines (partially cluster 8); eicosanoids (partly cluster 7, 17); and fatty acids (partly clusters 7, 8, 17) (Fig. 3a). Non-lipid clusters contained more structurally diverse metabolites, such as monosaccharides and nucleotide bases (partially clusters 8, 19), amino acids and peptides (partly clusters 1, 13, 15, 18, 20), and aromatic compounds (partly cluster 22) (Fig. 3a). A more detailed description of cluster composition is provided in Supplementary Fig. 5.

We next investigated associations between metabolite clusters using all metabolite pairs represented in at least 10 studies, applying aggregated Spearman’s correlation coefficients (see Methods “Correlation analysis between chemical classes” section for details). The analysis revealed strong positive correlations among most lipid clusters, whereas non-lipid clusters showed no consistent associations (Fig. 4a and b). The strongest correlations were generally observed between structurally related lipid clusters or within clusters themselves (e.g. clusters 6, 14, 23). Interestingly, non-lipid clusters did not exhibit strong self-correlations, in contrast to most lipid clusters. These findings indicate that structurally related metabolites tend to display coordinated abundances. The distinction between lipid and non-lipid clusters likely reflects the greater structural diversity within non-lipid groups (Supplementary Fig. 5). However, we could not exclude the possibility that difference in correlation patterns between lipids and non-lipids is associated with different chromatography and other technical issues, rather than biology. Nonetheless, not all strong correlations could be explained solely by structural similarity, and associations between non-related metabolite classes suggest possible biological or functional interactions that warrant further investigation (Fig. 4a and b).

**Figure 4:**
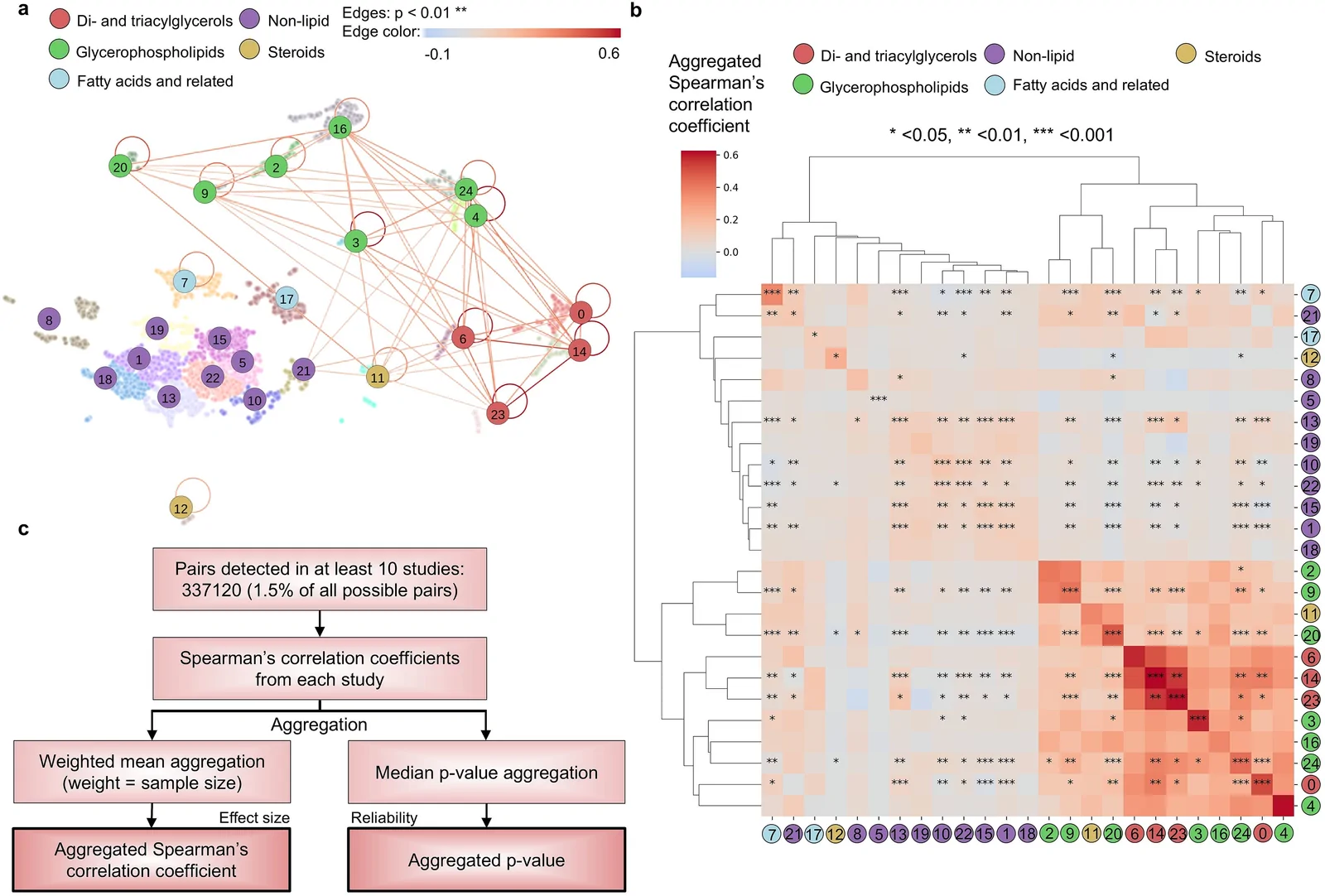
Structurally similar compounds tend to correlate quantitatively. (a) Graph representing the associations between chemical classes of metabolites. Edges are drawn if the aggregated *P*-value is less than 0.01. Edge color represents the aggregated Spearman’s correlation coefficient. Edge positions for each chemical class are determined by the localization of corresponding clusters on UMAP. (b) Heatmap showing the associations between chemical classes of metabolites. Color represents the aggregated Spearman’s correlation coefficient. Asterisks represent the aggregated *P*-value. (c) Detailed pipeline of the correlation analysis between chemical classes.

### Automated correlation analysis identifies metabolic correlates for clinically measured biomarkers

Metabolomics, with its broad coverage of small molecules in biofluids, offers a powerful approach for identifying novel disease biomarkers. However, most individual case–control studies are constrained by limited sample sizes, which restricts their ability to detect replicable associations. As an alternative strategy, we systematically screened all available Orbitrap- and QTOF-based human blood metabolomics studies on the Metabolomics Workbench to identify “metabolic correlates”—metabolites that exhibit the strongest correlations with clinically measured biomarkers based on several independent studies.

Using this automated large-scale approach, we examined several example biomarkers and identified the top three metabolites showing the strongest positive and negative correlations with each biomarker (Table 1). Among cardiovascular disease (CVD) biomarkers, we observed a strong association between homocysteine, an established independent risk factor for atherosclerosis [19], and linoleoyl-CoA. A previous metabolomics study of 75 blood samples reported that homocysteine is significantly linked to alterations in arachidonic acid and linoleic acid metabolism [20]. Linoleoyl-CoA serves as an intermediate in arachidonic acid biosynthesis from linoleic acid [21], and arachidonic acid and its metabolites are known to contribute to CVD risk through pro-inflammatory mechanisms [22]. Thus, the arachidonic acid pathway may provide a mechanistic link between homocysteinemia and CVD. Alternatively, linoleoyl-CoA may reflect linoleic acid oxidation [21], which lowers circulating linoleic acid levels promoting CVD [23]. If this is the case, increased dietary intake of linoleic acid could potentially mitigate CVD risk associated with elevated homocysteine.

In the context of cancer, we found strong correlations between pseudouridine, a recognized biomarker of prostate cancer [24], and two other modified uridines: 5,6-dihydrouridine and 3-(3-amino-3-carboxypropyl)uridine. Literature review confirmed that 5,6-dihydrouridine has been associated with multiple cancer types [25], including lung [26], liver [27], and prostate [28] cancer. In contrast, 3-(3-amino-3-carboxypropyl) uridine has not previously been reported as a cancer biomarker, making it a candidate for further investigation.

We also screened for metabolic correlates of creatinine, a standard biomarker for kidney disease [29]. Our findings supported an earlier metabolomics study reporting N,N,N-Trimethyl-alanylproline betaine as a novel biomarker of kidney function and dialytic clearance, outperforming creatinine [30].

### Open-source software tool to explore metabolite correlations

The automated screening approach demonstrated three key outcomes: it validated recently proposed biomarkers from individual studies, highlighted novel candidate biomarkers, and uncovered potential mechanistic links between established biomarkers and disease. Importantly, the large-scale search for metabolic correlates is not limited to the illustrative biomarkers presented here, it can be applied broadly across research contexts.

To facilitate this, we developed an open-source software tool that integrates Orbitrap- and QTOF-based human blood metabolomics data from the Metabolomics Workbench used in this study. The tool enables both hypothesis-driven and hypothesis-free analyses of metabolite associations (Supplementary Fig. 7). It consists of a main executable file launching Graphical User Interface (GUI) and accompanying JSON and SQLite database files containing precomputed correlations and metadata and can be run in three modes. In the first mode, users input two metabolite names and the tool outputs their correlations in individual studies and summarized statistics (Supplementary Fig. 7a). In the second mode, the tool outputs the strongest statistically significant (*P* < .05) positive and negative associations for a given metabolite with a user-defined thresholds for a minimal number of studies for metabolite to be included and number of displayed correlations (Supplementary Fig. 7b). In the third mode, it returns 1 to 10 (user-defined) most structurally similar compounds based on cosine similarity of SMILES embeddings (Supplementary Fig. 7c). Taken together, this software resource lowers the barrier to metabolomics data analysis for biologists and provides a practical framework for rapid hypothesis testing and discovery of novel metabolite associations.

### Curated meta-analysis identifies drug intake signatures in metabolomics data and reveals associated metabolic alterations

In addition to endogenous metabolites, metabolomics studies frequently detect exogenous compounds such as drugs, environmental pollutants, and other xenobiotics. These molecules are not consistently present in human biofluids and typically occur only in a subset of samples. However, our preliminary inspection of several drugs and their metabolites revealed that they were often reported at non-zero concentrations across the majority, or even all, samples within individual studies (Supplementary Fig. 8). Consequently, for many datasets, reported drug levels likely represent background noise rather than true exposure differences, rendering automated correlation analyses, and in particular based on relative rank as in Spearman, rather ineffective for such compounds. To overcome this limitation and extend our analytical framework to xenobiotics and other low-frequency metabolites, we conducted a manually curated meta-analysis focused on metabolomic changes associated with drug intake.

We automatically searched Orbitrap- and QTOF-based human blood studies on the Metabolomics Workbench for the presence of drugs and their metabolites and identified 77 studies in which a given drug appeared in at least five datasets (Fig. 5a). Each study was then manually reviewed to extract key metadata unavailable through automated parsing, including the true number of unique subjects (as opposed to total samples, which may include replicates or time points), details of data preprocessing and normalization, and available subject characteristics such as age, sex, and BMI. Following this curation, 24 studies met the inclusion criteria (Supplementary Table 1), encompassing 10 drugs: acetaminophen, metoprolol, atenolol, hydrochlorothiazide, naproxen, metformin, ibuprofen, ranitidine, gabapentin, and diphenhydramine, each represented in at least five studies (Fig. 5d).

**Figure 5:**
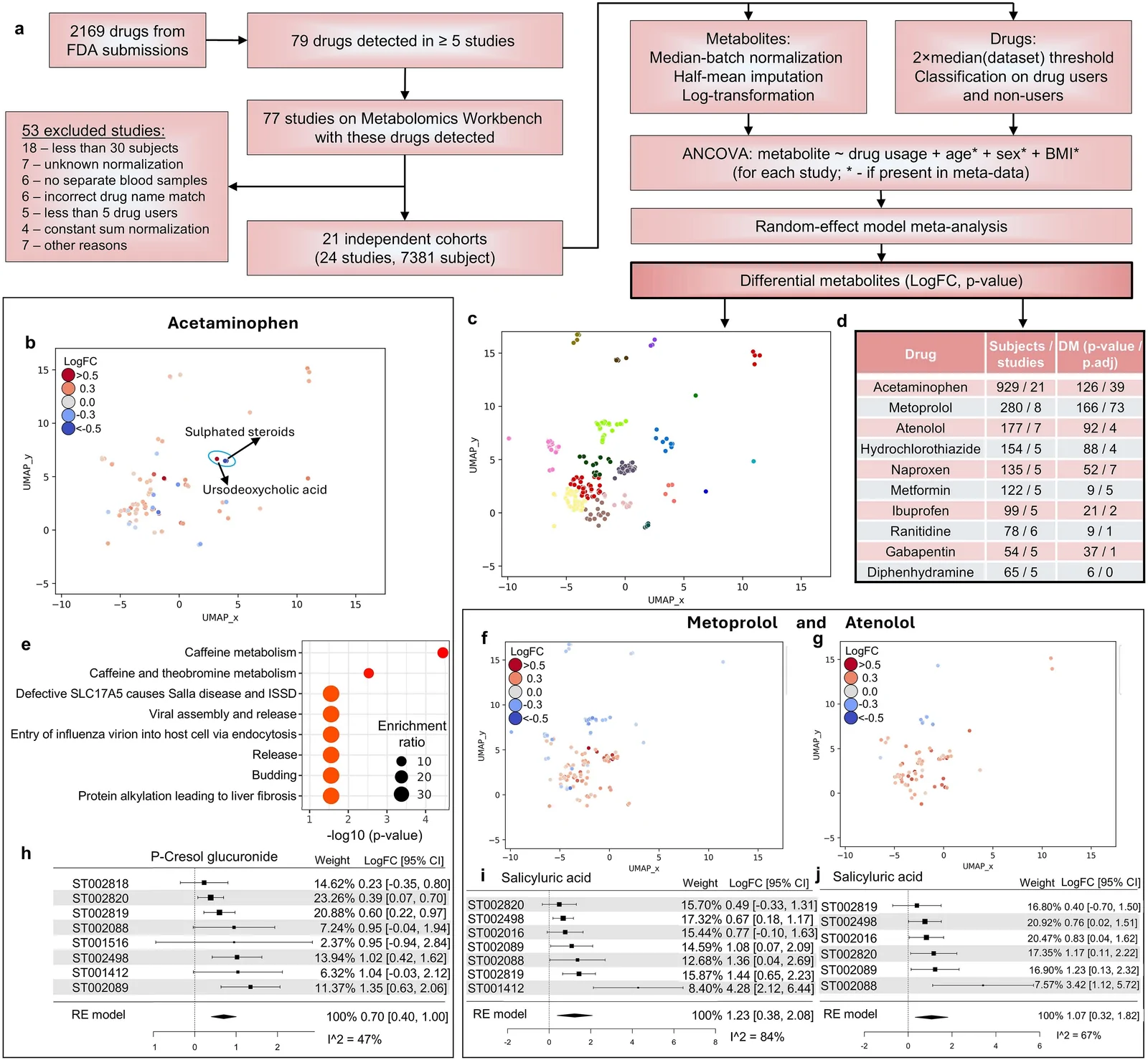
Curated meta-analysis of drug intake influence on metabolome. (a) Detailed pipeline of the curated meta-analysis on metabolic changes associated with drug intake. UMAPs showing metabolites associated with the use of acetaminophen (b), all tested drugs combined (c), metoprolol (f), and atenolol (g) with uncorrected *P* < .05. Metabolite positions are determined by the general UMAP for all 6752 metabolites. d table of meta-analysis results for 10 tested drugs presenting the information about the number of subjects and studies included in analyses and the number of differential metabolites (DM) by corrected (p.adj) and uncorrected (*P*-value) *P*-value threshold <.05. (e) Dot plot showing MetaboAnalyst enrichment analysis for metabolites positively associated with the acetaminophen intake (uncorrected *P* < .05). Forest plots demonstrating the meta-analysis results for the associations of acetaminophen with p-Cresol glucuronide (h), metoprolol with salicyluric acid (i), and atenolol with salicyluric acid (j).

We compared random-effects meta-analysis and mega-analysis approaches to assess robustness and found that the meta-analytic framework produced more conservative results (Supplementary Fig. 9). Therefore, we employed it for subsequent analyses. Differential metabolite associations with drug intake were summarized in Supplementary Tables 2–11 and visualized as forest plots for metabolites significant after FDR correction (Fig. 5h–j; Supplementary Figs 10–18). Enrichment analyses of significant metabolites were performed using MetaboAnalyst 6.0 (Supplementary Figs 20–29), and metabolite distributions were inspected in the previously generated UMAP space (Fig. 5c; Supplementary Fig. 19).

Across drugs, we observed a clear trend in which the number of differential metabolites decreased with smaller sample sizes, suggesting that most drug-specific meta-analyses remain underpowered (Fig. 5d). The acetaminophen dataset represented the largest and most statistically robust analysis, comprising ~1000 subjects across 20 studies. Consistent with previous findings [31], acetaminophen intake was strongly associated with decreased levels of sulfated steroids (Fig. 5b), including sulfated sex hormones and bile acids, a pattern that may underlie its reported adverse effects on reproductive health [31]. Enrichment analysis of metabolites elevated in acetaminophen users revealed associations with infection-related pathways and caffeine/theobromine metabolism, likely reflecting either disease itself or the common co-administration of caffeine-containing analgesics rather than direct pharmacological effects of acetaminophen.

We also identified metabolomic similarities between the β-blockers metoprolol and atenolol, which aggregated the largest sample size in meta-analysis after acetaminophen (Fig. 5d). Both drugs showed overlapping patterns of differential metabolites, such as salicylates (Fig. 5i–j), and shared enrichment of related biological processes (Supplementary Figs 21–22). Notably, metoprolol, and to a lesser extent atenolol, was associated with a significant reduction in circulating fatty acids (Fig. 5f–g), a finding consistent with the inhibition of peripheral lipolysis previously described for β-blockers [32]. Taken together, the curated meta-analysis of drug-associated metabolomic signatures successfully replicated known pharmacometabolomic effects from the literature while extending these findings to a broader dataset aggregated from the Metabolomics Workbench.

## Discussion

We present the first large-scale meta-analysis of publicly available metabolomics studies from the largest repository to date, the Metabolomics Workbench. By focusing on Orbitrap- and QTOF-based human blood datasets, we harmonized 182 studies encompassing 44 165 samples and 6752 unique metabolites after deduplication through cross-referencing with the PubChem database. This large-scale integration enabled a comprehensive characterization of the human blood metabolome, both structurally and quantitatively, and facilitated a systematic exploration of correlations between 25 chemical clusters based on associations in 337 120 metabolite pairs detected in ≥10 studies each. In addition, we performed targeted analyses to examine biomarker- and drug-associated metabolic alterations, which yielded 24 metabolic correlates for biomarkers including five with the correlation coefficient > 0.7 and 136 differential metabolites associated with drug intake after FDR-correction including 11 with |fold change| > 1.5, successfully reproducing several previously reported associations, thereby demonstrating the feasibility and power of large-scale meta-analyses across heterogeneous datasets from multiple small studies.

The largest metabolomics analyses published so far have primarily relied on the UK Biobank cohort, which includes over 250 000 participants analyzed by NMR spectroscopy [33–35]. Although the sample size in these studies is exceptional, the metabolite coverage remains limited to ~300 compounds, constrained by the analytical capacity of NMR. In contrast, MS based approaches can detect a far broader range of metabolites [36], but their application to large cohorts has so far been limited, with the largest MS-based studies including around 10 000 samples [37, 38]. To bridge these complementary strengths, we developed a framework for an underutilized yet powerful approach to performing meta-analysis of individual publicly available metabolomics studies.

Our study has several limitations. Although we restricted our analysis to Orbitrap- and QTOF-based human blood studies, considerable heterogeneity remained among the included datasets, due to cohort, sample handling, chromatography, ion mode, preprocessing, or normalization differences. To maintain the global scope of our analysis, we opted not to further constrain the dataset but instead relied on non-parametric statistical approaches to mitigate the impact of heterogeneity. Furthermore, except for the curated meta-analysis of drug intake, we were unable to verify whether all samples represented distinct individuals and correct the replicates and timepoints likely present in some datasets. We addressed this issue by leveraging the large overall sample size and sticking to non-parametrical statistics. Additionally, we did not analyze raw MS data and relied on metabolite names and abundances deposited by study authors. The metabolite identification is usually poorly described in Metabolomics Workbench metadata, and we were unable to verify its quality. In a curated meta-analysis of drug intake, it is hard to distinguish the effect of drug itself from other associated factors, such as disease, co-mediation, co-morbidity, etc., so the results should be interpreted with caution. Even though ChemBERTa appears to show decent performance it is possible that obtained chemical clusters from SMILES embeddings could be further improved if calculated directly from 3D molecule configurations. ChemBERTa has been trained only on a 77M-sized dataset, whereas SMILES formulas are non-unique per compound.

In conclusion, our work for the first time provides a proof of principle and a generalizable framework for large-scale meta-analysis of metabolomics data, demonstrated using Orbitrap- and QTOF-based human blood studies from the Metabolomics Workbench. The approach enables the combination of MS sensitivity yielding 6752 compounds in human blood with the largest for MS metabolomics up to date sample size of 44 165 samples aggregated from multiple individual studies. We anticipate that the adoption of this framework and the accompanying open-source software will accelerate hypothesis generation and validation in metabolomics, enabling researchers to take full advantage of the rapidly expanding volume of publicly available metabolomic data.

**Key Points**

- The presented framework enables large-scale mining of public metabolomics data and its re-analysis to obtain novel biomedical insights.
- The framework mined the data from 6752 human blood metabolites and the analysis of 337 120 metabolite pairs detected in at least 10 studies showed that structurally related clusters display correlated abundances (ρ up to 0.6).
- Targeted analyses for clinically relevant biomarkers and drugs revealed both known (e.g. homocysteine and linoleoyl-CoA, acetaminophen and sulfated steroids) and novel (e.g. 3-(3-amino-3-carboxypropyl)uridine) biomedical connections.

## Supplementary Material

Supplementary_materials_bbag576

## Data Availability

The analysed data are available at https://www.metabolomicsworkbench.org, while the code is available at https://github.com/FunctPharm-NeuroUU/metabolomics_workbench_meta-analysis.
