# Supplementary material for: Metabolomics workbench: data-driven classification and correlation analysis between metabolite classes, biomarkers, and drugs
Source: Brief Bioinform. 2026 Oct 19;27(5):bbag576. doi: 10.1093/bib/bbag576 (PMC13677790; doi:10.1093/bib/bbag576)
Supplement: Supplementary file 1 [file supplementary_materials_bbag576.docx]

**Supplementary Information**

**Extended Methods**

**Study selection**

Metadata containing study identifiers and species information for all studies in the Metabolomics Workbench repository was retrieved on June 7, 2025, using the Metabolomics Workbench REST API (Representational State Transfer Application Programming Interface) and the Python libraries *requests* and *json*.

Because the primary aim of this study was to enable large-scale reanalysis of metabolomics data for biomedical research, we focused exclusively on human studies. Filtering for human studies across all publicly available data yielded 1,407 studies (**Fig. 1**, **Supplementary Fig. 1a**). For each study, we automatically retrieved key technical characteristics, including analysis type (mass spectrometry [MS] or nuclear magnetic resonance [NMR]), MS instrument type, chromatography type, sample type, and units of measurement, using the same REST API.

To ensure comparability across studies, we restricted our analysis to blood-based studies, by far the most common sample type in the repository (**Supplementary Fig. 1c**), that used the two most frequently represented in Metabolomics Workbench MS instrument types: Orbitrap and QTOF (**Supplementary Fig. 1b**). Applying these filters resulted in 291 studies, 109 of which did not contain named metabolite data. To ensure reproducibility and avoid raw MS data annotation steps, we limited our analysis to studies with already annotated metabolite data. The final dataset consisted of 182 studies, which were used for all subsequent analyses.

**Data retrieval**

Metabolomics Workbench has an internal unified metabolite name system ascribing special RefMet names to almost all metabolites in a study, which helps to prevent the issue of a single metabolite being named by different synonyms in different studies. However, as one of our aims was to perform structural embedding and clustering based on SMILES (Simplified Molecular Input Line Entry System, line notation for chemical structure), which is not reported in many cases in Metabolomics Workbench metadata, we decided to retrieve SMILES from the PubChem database. Therefore, our first task was to map RefMet names or metabolite names given by study authors to PubChem CIDs.

As a first step, we extracted the Metabolomics Workbench metadata for all RefMet metabolites and constructed a dictionary for those with available PubChem CID. Then, for each metabolite in each study, typically represented by an author-provided name and a RefMet name (although the latter was sometimes missing), we first checked whether a PubChem CID was available in the Metabolomics Workbench metadata. If not, we attempted to retrieve the PubChem CID from the PubChem database using the RefMet name as a query, and, if that was unavailable or did not yielded the PubChem CID, using the author-provided metabolite name. This procedure yielded 6,138 PubChem CIDs. After that, we noticed that the PubChem CID retrieval failed mostly for lipid metabolites, whose RefMet names are given in a form of abbreviations (e.g., *PC 16:0/18:1*) not recognized by PubChem. To address this, we performed an additional search by RefMet names in the LIPID MAPS database, retrieving 679 PubChem CIDs. After removing duplicates, we obtained 6,752 unique PubChem CIDs from an initial pool of 6,779 RefMet names associated with 28,841 author-reported metabolite names. SMILES strings were then retrieved for each CID from PubChem using the REST API and the Python libraries *requests* and *json*.

**Metabolite structural embedding by SMILES**

Structural embedding of metabolites was performed using the ChemBERTa-77M-MTR model and tokenizer, loaded via Python *transformers* library. The 6,752 SMILES strings associated with previously retrieved PubChem CIDs were embedded and the resulting 600-dimentional vectors for each SMILES were subjected to dimension reduction using Principal Component Analysis (PCA) and Uniform Manifold Approximation and Projection (UMAP), implemented with Python libraries *sklearn* and *umap*, respectively. PCA and UMAP have been chosen as common examples of linear and non-linear methods for dimension reduction, respectively.

**Metabolite structural clustering**

We aimed to perform structural clustering of metabolites based on SMILES embeddings and compared several clustering methods: k-means on PCA-reduced embeddings, bisecting k-means on PCA-reduced embeddings, agglomerative (hierarchical) clustering on PCA-reduced embeddings, and k-means on UMAP-reduced embeddings. The number of PCA components for clustering was determined by the elbow method (**Supplementary Fig. 3a**) and set to 7. Two UMAP components were used for clustering. We set the number of clusters to 25 for all clustering methods, based on the approximate number of separate clouds on the UMAP plot. All clustering methods were conducted using the Python library *sklearn*.

To evaluate clustering performance, we retrieved chemical class annotations using ClassyFire web service (**Supplementary Fig. 3b, c**). As better clustering would generate more homogenous clusters in terms of the present main metabolite classes, we quantified cluster homogeneity using the Gini impurity metric (1)

$Gini impurity = 1 - \Sigma p_{i}^{2}$ (1)

where *p_i_* is the frequency of the *i^th^* main metabolite class within a cluster. Gini impurity was calculated for each cluster and then averaged to produce an overall clustering quality metric.

Despite concerns about using UMAP-reduced data for clustering, the evaluation demonstrated that k-means clustering on UMAP-reduced embeddings in our particular case produced the most homogeneous clusters (mean Gini impurity = 0.737) compared with all PCA-based methods (mean Gini impurity = 0.876, 0.894, 0.865; Supplementary Fig. 4). Based on these results, we selected k-means clustering on UMAP-reduced data for all subsequent analyses.

**Correlation analysis between chemical classes**

After clustering metabolites into 25 chemical clusters, we aimed to estimate the correlations between these clusters. Given the heterogeneity of the included studies, including differences in cohorts, instrumentation, and measurement units, we decided to use the nonparametric approach using Spearman’s rank correlation coefficient. Correlations between clusters were derived by aggregating all available metabolite–metabolite correlations for a cluster pair across individual studies.

To perform this correlation analysis, we first extracted study-level metadata (number of samples and number of metabolites) and quantitative metabolite data for each sample using the Metabolomics Workbench REST API and the Python libraries *requests* and *json*. Missing values within each study were imputed with half of the minimum reported value for that study. This method (present in forms of minimum value imputation or half minimum imputation) has been chosen as one of the most popular in large metabolomics studies on Metabolomics Workbench to maximize homogeneity in data processing. Using the imputed data, we computed pairwise Spearman’s correlation coefficients and corresponding p-values for all metabolites within each study using the Python library *scipy*.

To estimate the correlation between two chemical clusters (cluster₁, cluster₂), we refined all metabolite pairs (metabolite₁ ∈ cluster₁, metabolite₂ ∈ cluster₂) that were reported in at least 10 studies. For each metabolite pair, individual Spearman’s correlation coefficients were aggregated by calculating a weighted mean of primary Spearman’s correlations per study (with sample sizes in studies being the weights) and p-values were aggregated by taking a median p-value of individual primary p-values (metabolite-level aggregation):

$\rho_{metabolite pair} = \frac{\sum_{i = 1}^{n} \rho_{study i} \times{sample size}_{i}}{\sum_{i = 1}^{n} {sample size}_{i}}$ (2)

${p-value}_{metabolite pair} = median({p-value}_{individual studies})$ (3)

To calculate the final estimate of correlation between chemical clusters, aggregated Spearman’s correlation coefficients were aggregated for the second time by calculating a weighted mean (with sums of sample sizes in studies being the weights) and p-values were aggregated by taking a median (cluster-level aggregation):

$\rho_{cluster pair} = \frac{\sum_{i = 1}^{n} \rho_{metabolite pair i} \times sum {sample size}_{i}}{\sum_{i = 1}^{n} sum {sample size}_{i}}$ (4)

${p-value}_{cluster pair} = median({p-value}_{metabolite pairs})$ (5)

The estimates of intracluster correlation were also calculated using the same approach but restricting both metabolites to the same cluster.

Finally, we performed sensitivity analysis of the aggregation method by comparing results obtained using weighted mean, unweighted mean, and median aggregation of Spearman’s coefficients (**Supplementary Fig. 6**). The results were robust, showing no significant differences across aggregation methods.

**Automated screening for metabolite associations**

To enable access to Metabolomics Workbench data and facilitate rapid hypothesis testing of metabolite associations and the identification of potential metabolic correlates or structurally similar compounds, we developed a specialized software tool (see Code availability section). This script utilizes Spearman’s correlation data for all possible metabolite pairs present in the studies analyzed in this work. The correlation data are contained in 5 JSON and 1 SQLite database files, which should be placed in the directory ./data from the main executable file “Metabolite Correlation Explorer.exe” for the proper work

The script can be run in three modes: the first is for validating hypotheses on metabolite associations, the second is for screening for potential metabolic correlates, and the third is for retrieving the most structurally similar compounds. To run the first mode the user is required to provide 2 metabolite names as input, and the information about correlation between these metabolites will be output (**Supplementary Fig. 7a**). Aggregation of individual Spearman’s correlation coefficients is performed by calculating a weighted mean (with sample sizes in studies being the weights), and p-values are aggregated by taking a median, but a detailed table of per-study correlation coefficients and p-values is also returned. In the second mode, it outputs the strongest significant (p < 0.05) positive and negative associations for a given metabolite (**Supplementary Fig. 7b**). Aggregation of individual Spearman’s correlation coefficients is performed by calculating a weighted mean (with sample sizes in studies being the weights), and p-values are aggregated by taking a median. These aggregated metrics for each metabolite pair are then used to perform thresholding (by p-value < 0.05) and sorting (by aggregated Spearman’s correlation coefficient), generating the output of strongest significant positive and strongest significant negative metabolic correlates for a given metabolite. In the third mode, it outputs the most structurally similar compounds based on the cosine similarity between SMILES embeddings (**Supplementary Fig. 7c**).

This automated screening tool in the second mode was used to generate the results presented in **Table 1**, which lists the metabolic correlates for clinically measured biomarkers.

**Study screening for drug intake association analysis**

Although the automated correlation approach described in section “Automated screening for metabolite associations” enables rapid hypothesis testing, it should be applied with caution – particularly for rare metabolites, drugs, and xenobiotics. For example, when searching for metabolites associated with drug intake, we found that the reported drug concentrations in some studies likely represented mainly background noise, as it is improbable for a particular drug to appear in nearly all population-based biological samples in a study. This pattern was observed across multiple datasets. Therefore, for rare metabolites, drugs, xenobiotics, etc., which are naturally present only in part of samples, the developed approach of non-parametric automatic screening will not be suitable, as the correlation would be driven mostly by background data. To address this limitation, we performed a highly curated meta-analysis of metabolites associated with drug intake.

As an initial set of studies, we used the same 1,407 human blood-based studies analyzed on Orbitrap or QTOF instruments that were refined previously (see section “Study selection”). We then automatically screened these studies for the presence of drugs or drug metabolites among the annotated compounds.

To perform this automatic screening, we extracted drug names from the FDA submissions data, automatically excluded several general terms ("sodium", "potassium", "acetate", "sulfate", "phosphate", "hydrochloride", "calcium", "nitrate", "maleate", "fumarate", "bromide", "citrate", "succinate", "iodide", "chloride") from them, and corrected drug names manually to retain drug specific words for the search. These truncated drug names were matched with the DrugBank synonym names data using the drug_table_parsing.R script. Finally, truncated drug names and their synonyms from DrugBank were used to automatically retrieve the studies containing the drugs as annotated compounds: we found 79 drugs present in at least 5 studies and a total of 77 studies containing them.

The resulting 77 studies were screened manually, which allowed us to collect the information about the actual number of subjects (in contrast to the automatically accessible number of samples that may be technical replicates or timepoints related to the single subject), data processing and normalization procedures, and subject metadata (e.g., age, sex, BMI). As a result of the screening, 24 studies were included in the analysis (**Supplementary Table 1**). The primary exclusion reasons were the number of subjects fewer than 30, unknown or inappropriate normalization procedures, absence of separate blood samples in the data, and incorrect drug name match.

For the included 24 studies, data deposited on the Metabolomics Workbench were retrieved and separated into two datasets: drugs (containing drug and drug-metabolite measurements) and non-drug (containing all other metabolites) using a list of drug names generated previously. The datasets of drugs and non-drug metabolites along with the subject metadata (e.g., age, sex, BMI) datasets for each study were saved for further processing using R scripts.

**Meta-analysis and mega-analysis of metabolites associated with drug intake**

To harmonize the studies for meta-analysis and mega-analysis and ensure that all samples included in the analysis are actual biological replicates from independent subjects, several preprocessing stages were performed (dataset_preprocessing_1.R). In studies ST002553 and ST002301, technical replicates for each subject were aggregated by taking mean metabolite concentrations. Quality control samples in the study ST002016 were removed from further analysis. ST001236 and ST001236 studies were converted back from log-scale. Timepoints other than baseline were omitted for studies ST000105, ST000695, ST000974, ST000975, ST001236, ST001237, ST001516, ST001517, and ST001849. As we aimed to cover the maximal number of metabolites, separate datasets from different ion modes and chromatography columns were merged if multiple were present for a single study.

All the datasets containing raw data were normalized using the median-batch method (all concentrations were divided by the median value for the whole batch or whole dataset, if batch data were not present in the metadata). All normalized datasets included in the analysis also used median-batch normalization. Missing values were imputed with the half minimal value for the metabolite; metabolites containing only missing values were removed from the datasets. Then we log-transformed median-batch normalized datasets and combined studies ST001236 with ST001237, ST001516 with ST001517, and ST000974 with ST000975 that contained patients from the same cohorts. Thus, we generated similarly processed (median-batch normalization, half-minimum imputation, log-transformation) datasets from 21 independent cohorts for further analysis.

Datasets containing drug data were processed separately (dataset_preprocessing_2.R, dataset_preprocessing_3.R). All metabolite forms of a single drug, as well as all technical replicates, were averaged to yield a single estimate of drug usage. As most of the subjects with the detected drug concentration (which, for some datasets and drugs, were detected in all samples) were actual non-takers, we set a task to create a threshold to classify subjects as drug takers and non-takers. We selected a value twice the dataset median as a suitable threshold based on the examination of multiple cases of its performance by visual inspection of distribution patterns (**Supplementary Fig. 8**). The cases were coherent with the intuition of concentration of drug non-takers forming a peak in low values, and few actual drug takers being in the high value zone. Finally, we merged processed datasets of binary drug intake data and non-drug metabolite concentrations, and added cofounders (sex, age, and BMI, if present) from metadata (dataset_preprocessing_4.R). We decided to perform the analysis for drugs present in at least 5 cohorts, which refined 10 drugs: acetaminophen, metoprolol, atenolol, hydrochlorothiazide, naproxen, metformin, ibuprofen, ranitidine, gabapentin, and diphenhydramine.

To aggregate the data between studies, we decided to test two approaches: random-effects meta-analysis and mega-analysis. For meta-analysis, in each study we tested the association between concentrations of metabolites and drug intake (independent variable) by an ANCOVA model (implemented by the R package *stats*) with age, sex, and BMI included in the model as covariates, if present in the data. Then LogFC (calculated from adjusted means computed by the R package *lsmeans*[^61^](https://www.zotero.org/google-docs/?WWj3wP)) and p-values for each metabolite and for each study were meta-analyzed by a random-effect model. Heterogeneity was estimated using Sidik-Jonkman estimator (implemented by the R package *metafor*[^62^](https://www.zotero.org/google-docs/?lEnPal)), if there were at least 5 studies with the tested pair drug – metabolite. Study weight was determined by the inverse variance method (*metafor*'s default). Sidik-Jonkman estimator[^63^](https://www.zotero.org/google-docs/?EWx7IR) was selected as we expected high heterogeneity between studies, and such estimator generally performs well in high heterogeneity and has positive bias in low heterogeneity, keeping the analysis rather conservative. Additionally, it has a closed-form solution, ensuring computational speed and guaranteed "convergence".

MS instrument type was included as a moderator if there were at least 3 studies with each instrument type (Orbitrap and QTOF). In parallel, a mega-analysis was performed in which subjects from all studies for a particular metabolite were combined in one ANCOVA model (implemented by R packages *stats* and *lsmeans*) , with study and instrument type (Orbitrap and QTOF) were included in a model as confounders. We intersected the results of the meta-analysis and mega-analysis using the same FDR-corrected p-value threshold (< 0.05) and found that, except for one metabolite identified by the meta-analysis, all differential metabolites were also detected in the mega-analysis. The mega-analysis additionally identified more differential metabolites (**Supplementary Fig. 9**). Therefore, the meta-analysis turned out to be a more conservative approach, and we decided to use its results for further analysis. We used lists of differential metabolites filtered by an uncorrected p-value threshold < 0.05 for enrichment analysis and plotting on structural UMAP to broaden the specter of metabolites and be able to detect global trends in the results; however, we present differential metabolites with both corrected and uncorrected p-values in **Supplementary Tables 2-11**.

**Functional annotation by MetaboAnalyst**

Using the nominally significant (uncorrected p-value < 0.05) differential metabolites found by meta-analysis, functional annotation was performed in MetaboAnalyst 6.0 web-service. RaMP-DB sets were used for enrichment analysis, and the reference library was constructed from all metabolites detected across the included studies.

**Supplementary Figure 1. Metabolomics Workbench data content. A** The distribution of all studies on Metabolomics Workbench (on June 7, 2025) by species. **B** The distribution of human studies on Metabolomics Workbench by instrument type used for detection. **C** The distribution of human studies on Metabolomics Workbench by sample type. **D** The distribution of 6752 PubChem CIDs retrieved from 1407 blood-based human studies from Metabolomics Workbench using Orbitrap or QTOF for detection by the number of studies in which these CIDs were reported.

**Supplementary Figure 2. Quantitative comparison between Orbitrap and QTOF instruments.** UMAPs generated on the SMILES embeddings by the ChemBERTa model colored by the median percentile of the metabolite concentration among all detected metabolites in Orbitrap (**A**) and QTOF (**B**) studies.

**Supplementary Figure 3. Determination of clustering parameters and exploration of validation data. A** The elbow plot for Principal Component Analysis (PCA) demonstrates variance explained by each principal component. Based on this plot the number of principal components for clustering was set to 7. **B** The distribution of 6752 PubChem CIDs retrieved from 1407 blood-based human studies from Metabolomics Workbench using Orbitrap or QTOF for detection by the main metabolite class from PubChem metadata. **C** UMAPs generated on the SMILES embeddings by the ChemBERTa model colored by the main metabolite class from PubChem metadata.

**Supplementary Figure 4. Comparison of clustering performance by tested methods.** UMAPs generated on the SMILES embeddings by the ChemBERTa model colored by the cluster number for the k-means clustering on PCA-reduced embeddings (**A**), k-means clustering on UMAP-reduced embeddings (**B**), bisecting k-means clustering on PCA-reduced embeddings (**C**), and hierarchal clustering on PCA-reduced embeddings (**D**). Homogeneity of clusters were assessed using mean Gini impurity metric of all clusters (Gini impurity=1−∑p_i_^2^, where *p_i_* is the frequency of the *i^th^* main metabolite class from PubChem metadata within a cluster).

**Supplementary Figure 5. Annotation of the UMAP with clusters from k-means on UMAP-reduced embeddings by main metabolite class metadata from PubChem.** Numerators of the fraction are numbers of metabolites from a particular main metabolite class in a cluster, while denominators are the total number of metabolites in the cluster.

**Supplementary Figure 6. Sensitivity analysis on aggregation methods for Spearman’s correlation coefficients.** Heatmaps show correlations between clusters aggregated by weighted mean (**A**), unweighted mean (**B**), and median (**C**). Weights are sample sizes in studies. Aggregated (by median) p-values of the correlations are denoted as * (<0.05), ** (<0.01), and *** (<0.001).

**Supplementary Figure 7. Developed open-source software tool that enables correlation analysis and search for metabolic correlates or structurally similar compounds for any metabolite in the explored Metabolomics Workbench data.** The script can be run in three modes: the first takes 2 metabolite names as input and serves for validating hypotheses on metabolite associations outputting aggregated and individual Spearman’s correlation coefficients and p-values for these two metabolites (**A**); the second takes only one metabolite name and output the strongest significant (p < 0.05) positive and negative associations for a given metabolite (**B**), and the third takes one metabolite and outputs most structurally similar compounds (**C**).

**Supplementary Figure 8. Drug concentration thresholding with twice dataset median value.** Representative example plots of the acetaminophen (**A**), metoprolol (**B**), atenolol (**C**), and hydrochlorothiazide (**D**) distributions with twice dataset median threshold represented as a vertical line. All subjects right to the line were defined as drug takers, while left to the line – as non-takers (background signal). Concentrations of acetaminophen (**A**) and hydrochlorothiazide (**D**) are given in median-batch normalized values in the presented studies, while concentrations of metoprolol (**B**) and atenolol (**C**) are given in raw values. Study IDs are located in top right corners.

**Supplementary Figure 9. Intersection of the meta-analysis and mega-analysis results on associations of metabolites with drug intake.** Euler diagrams for each of the 10 tested drugs demonstrate the number of differential metabolites (with FDR-adjusted p-value < 0.05) found by meta-analysis and mega-analysis. The total number of tested metabolites (that was reported in at least 5 studies with the tested drug) are given in parenthesis.

**Supplementary Figure 10. Differential metabolites associated with acetaminophen intake.** Forest plots with meta-analysis results on metabolites associated with acetaminophen intake (FDR-corrected p-value < 0.05).

**Supplementary Figure 11. Differential metabolites associated with metoprolol intake.** Forest plots with meta-analysis results on metabolites associated with metoprolol intake (FDR-corrected p-value < 0.05).

**Supplementary Figure 12. Differential metabolites associated with atenolol intake.** Forest plots with meta-analysis results on metabolites associated with atenolol intake (FDR-corrected p-value < 0.05).

**Supplementary Figure 13. Differential metabolites associated with hydrochlorothiazide intake.** Forest plots with meta-analysis results on metabolites associated with hydrochlorothiazide intake (FDR-corrected p-value < 0.05).

**Supplementary Figure 14. Differential metabolites associated with naproxen intake.** Forest plots with meta-analysis results on metabolites associated with naproxen intake (FDR-corrected p-value < 0.05).

**Supplementary Figure 15. Differential metabolites associated with metformin intake.** Forest plots with meta-analysis results on metabolites associated with metformin intake (FDR-corrected p-value < 0.05).

**Supplementary Figure 16. Differential metabolites associated with ibuprofen intake.** Forest plots with meta-analysis results on metabolites associated with ibuprofen intake (FDR-corrected p-value < 0.05).

**Supplementary Figure 17. Differential metabolites associated with ranitidine intake.** Forest plots with meta-analysis results on metabolites associated with ranitidine intake (FDR-corrected p-value < 0.05).

**Supplementary Figure 18. Differential metabolites associated with gabapentin intake.** Forest plots with meta-analysis results on metabolites associated with gabapentin intake (FDR-corrected p-value < 0.05).

**Supplementary Figure 19. UMAPs of drug intake associated metabolites.** UMAPs showing metabolites associated with the use of hydrochlorothiazide (A), naproxen (B), metformin (C), ibuprofen (D), ranitidine (E), gabapentin (F), and diphenhydramine (G) with uncorrected p-value < 0.05. Metabolite positions are determined by the general UMAP for all 6752 metabolites.

**Supplementary Figure 20. MetaboAnalyst enrichment for acetaminophen.** Dot plot showing MetaboAnalyst enrichment analysis for metabolites positively (**A**) and negatively (**B**) associated with the acetaminophen intake (uncorrected p-value < 0.05).

**Supplementary Figure 21. MetaboAnalyst enrichment for metoprolol.** Dot plot showing MetaboAnalyst enrichment analysis for metabolites positively (**A**) and negatively (**B**) associated with the metoprolol intake (uncorrected p-value < 0.05).

**Supplementary Figure 22. MetaboAnalyst enrichment for atenolol.** Dot plot showing MetaboAnalyst enrichment analysis for metabolites positively (**A**) and negatively (**B**) associated with the atenolol intake (uncorrected p-value < 0.05).

**Supplementary Figure 23. MetaboAnalyst enrichment for hydrochlorothiazide.** Dot plot showing MetaboAnalyst enrichment analysis for metabolites positively associated with the hydrochlorothiazide intake (uncorrected p-value < 0.05).

**Supplementary Figure 24. MetaboAnalyst enrichment for naproxen.** Dot plot showing MetaboAnalyst enrichment analysis for metabolites positively (**A**) and negatively (**B**) associated with the naproxen intake (uncorrected p-value < 0.05).

**Supplementary Figure 25. MetaboAnalyst enrichment for metformin.** Dot plot showing MetaboAnalyst enrichment analysis for metabolites positively (**A**) and negatively (**B**) associated with the metformin intake (uncorrected p-value < 0.05).

**Supplementary Figure 26. MetaboAnalyst enrichment for ibuprofen.** Dot plot showing MetaboAnalyst enrichment analysis for metabolites positively (**A**) and negatively (**B**) associated with the ibuprofen intake (uncorrected p-value < 0.05).

**Supplementary Figure 27. MetaboAnalyst enrichment for ranitidine.** Dot plot showing MetaboAnalyst enrichment analysis for metabolites positively (**A**) and negatively (**B**) associated with the ranitidine intake (uncorrected p-value < 0.05).

**Supplementary Figure 28. MetaboAnalyst enrichment for gabapentin.** Dot plot showing MetaboAnalyst enrichment analysis for metabolites positively (**A**) and negatively (**B**) associated with the gabapentin intake (uncorrected p-value < 0.05).

**Supplementary Figure 29. MetaboAnalyst enrichment for diphenhydramine.** Dot plot showing MetaboAnalyst enrichment analysis for metabolites positively associated with the diphenhydramine intake (uncorrected p-value < 0.05).

**Supplementary Table 1. Studies included in meta-analysis of metabolites associated with drug intake.**

| **Study ID** | **Number of subjects** | **Number of drugs** | **MS instrument** | **Units of measurement** | **Cohort** |
| --- | --- | --- | --- | --- | --- |
| ST002089 | 1125 | 42 | Orbitrap | median batch-normalized peak area | COPDGene (COPD patients) |
| ST002820 | 1002 | 28 | Orbitrap | median batch-normalized peak area | MDRD: The Modification of Diet in Renal Disease study |
| ST002819 | 751 | 32 | Orbitrap | median batch-normalized peak area | AASKG1: African American Study of Kidney |
| ST001237 | 741 | 9 | Orbitrap | peak area | Patients with metastatic renal cell carcinoma (RCC) |
| ST002088 | 648 | 41 | Orbitrap | median batch-normalized peak area | SPIROMICS (COPD patients) |
| ST002498 | 580 | 40 | Orbitrap | peak area | Patients who underwent prostate biopsy |
| ST001849 | 322 | 3 | QTOF | peak area | Tested for COVID19 |
| ST002818 | 289 | 22 | Orbitrap | median batch-normalized peak area | ALTOLD: Assessing Long Term Outcomes in Living Kidney Donors study |
| ST000974 | 216 | 5 | Orbitrap | median batch-normalized peak area | GC6-74 cohorts (4,462 HIV-negative healthy household contacts of 1,098 index TB cases) at four African sites |
| ST003520 | 210 | 12 | Orbitrap | peak area | Crohn's Disease, Juvenile Idiopathic Arthritis, controls |
| ST000946 | 207 | 5 | QTOF | peak area | ELEMENT (early life toxin exposure) |
| ST000975 | 177 | 6 | Orbitrap | median batch-normalized peak area | GC6-74 cohorts (4,462 HIV-negative healthy household contacts of 1,098 index TB cases) at four African sites |
| ST002016 | 140 | 17 | Orbitrap | peak area | Tested positive for COVID19 |
| ST000919 | 139 | 1 | Orbitrap | peak height | Fasting plasma samples collected at baseline |
| ST001412 | 131 | 31 | Orbitrap | median batch-normalized peak area | Obese patients with and without neuropathy, lean controls |
| ST001828 | 100 | 1 | QTOF | peak area | Pregnancy cohort (preterm birth and controls) |
| ST001516 | 99 | 5 | Orbitrap | peak area | Cohort of West African children, collected before and during P. falciparum malarial infection |
| ST001236 | 91 | 9 | Orbitrap | peak area | Patients with metastatic renal cell carcinoma (RCC) |
| ST002553 | 88 | 1 | QTOF | peak area | Chronic renal failure (CRF) patients |
| ST002301 | 85 | 1 | QTOF | peak area | Tested positive for COVID19 |
| ST000992 | 79 | 1 | QTOF | peak area | Case-control study of myocardial infarction in Costa Rican adults |
| ST000105 | 58 | 3 | QTOF | peak area | Polycystic ovary syndrome (PCOS) women w/ and w/o OSA |
| ST001517 | 53 | 3 | Orbitrap | peak area | Cohort of West African children, collected before and during P. falciparum malarial infection |
| ST000695 | 50 | 4 | QTOF | peak area | Patients before and after aromatase inhibitor therapy |

**Supplementary Table 2. Differential metabolites found by meta-analysis to be associated with acetaminophen intake.** Bold font represents metabolites remaining significantly associated (p<0.05) after FDR correction. MS instrument was included as a moderator in meta-analysis when there were at least three studies with each instrument type (Orbitrap and QTOF).

| drug | metabolite | LogFC | SE | p_val | p.adj | number of studies | I2 | Moderator logFC | Moderator p_val |
| --- | --- | --- | --- | --- | --- | --- | --- | --- | --- |
| **acetaminophen** | **P-Cresol glucuronide** | **0.701** | **0.155** | **5.96E-06** | **6.74E-04** | **8** | **47** | **NA** | **NA** |
| **acetaminophen** | **Salicyluric glucuronide** | **0.517** | **0.161** | **1.37E-03** | **3.43E-02** | **11** | **78** | **NA** | **NA** |
| acetaminophen | Ursodeoxycholic acid | 0.496 | 0.166 | 2.76E-03 | 5.94E-02 | 11 | 66 | 0.786 | 0.036 |
| **acetaminophen** | **5-Acetylamino-6-amino-3-methyluracil** | **0.477** | **0.131** | **2.74E-04** | **1.19E-02** | **10** | **26** | **NA** | **NA** |
| acetaminophen | Maltose | 0.380 | 0.169 | 2.47E-02 | 2.49E-01 | 9 | 77 | NA | NA |
| **acetaminophen** | **1,7-Dimethyluric acid** | **0.376** | **0.122** | **2.16E-03** | **4.90E-02** | **14** | **34** | **NA** | **NA** |
| acetaminophen | Caffeine | 0.355 | 0.154 | 2.15E-02 | 2.34E-01 | 20 | 68 | 0.034 | 0.927 |
| acetaminophen | CAR DC8:0 | 0.349 | 0.117 | 2.74E-03 | 5.94E-02 | 8 | 69 | NA | NA |
| acetaminophen | Dimethylphenol sulfate | 0.344 | 0.140 | 1.44E-02 | 1.78E-01 | 6 | 39 | NA | NA |
| acetaminophen | 7-Methyluric acid | 0.330 | 0.161 | 4.11E-02 | 3.18E-01 | 5 | 62 | NA | NA |
| acetaminophen | 1,3,7-Trimethyluric acid | 0.329 | 0.153 | 3.17E-02 | 2.79E-01 | 9 | 66 | NA | NA |
| **acetaminophen** | **Indole-3-methyl acetate** | **0.327** | **0.099** | **9.60E-04** | **2.63E-02** | **12** | **40** | **-0.452** | **0.106** |
| acetaminophen | DG 18:2_20:4 | 0.313 | 0.151 | 3.78E-02 | 3.07E-01 | 6 | 50 | NA | NA |
| **acetaminophen** | **CAR DC5:0;3Me** | **0.304** | **0.084** | **2.84E-04** | **1.19E-02** | **10** | **39** | **NA** | **NA** |
| **acetaminophen** | **3-Methyladipic acid** | **0.301** | **0.085** | **4.15E-04** | **1.56E-02** | **9** | **54** | **NA** | **NA** |
| acetaminophen | N2-Acetyl,N6,N6-dimethyllysine | 0.294 | 0.144 | 4.09E-02 | 3.18E-01 | 5 | 6 | NA | NA |
| acetaminophen | Imidazole propanoate | 0.289 | 0.100 | 3.77E-03 | 7.93E-02 | 11 | 62 | NA | NA |
| **acetaminophen** | **N-Acetylkynurenine** | **0.289** | **0.061** | **2.36E-06** | **5.33E-04** | **5** | **6** | **NA** | **NA** |
| acetaminophen | 3-Hydroxy-adipic acid | 0.289 | 0.141 | 3.99E-02 | 3.15E-01 | 7 | 60 | NA | NA |
| **acetaminophen** | **MG 18:1/0:0/0:0** | **0.281** | **0.069** | **4.51E-05** | **3.14E-03** | **7** | **38** | **NA** | **NA** |
| acetaminophen | CAR DC6:0 | 0.274 | 0.103 | 7.81E-03 | 1.28E-01 | 9 | 67 | NA | NA |
| acetaminophen | N-methyl pipecolic acid | 0.273 | 0.099 | 5.62E-03 | 1.04E-01 | 8 | 31 | NA | NA |
| acetaminophen | PE 16:0/22:6 | 0.273 | 0.102 | 7.35E-03 | 1.28E-01 | 5 | 43 | NA | NA |
| acetaminophen | CAR DC7:0 | 0.273 | 0.132 | 3.79E-02 | 3.07E-01 | 8 | 77 | NA | NA |
| **acetaminophen** | **Sorbitol** | **0.270** | **0.083** | **1.20E-03** | **3.18E-02** | **10** | **26** | **NA** | **NA** |
| acetaminophen | N-Phenylacetylglutamic acid | 0.269 | 0.118 | 2.31E-02 | 2.42E-01 | 8 | 63 | NA | NA |
| acetaminophen | N-Acetyl-3-methylhistidine | 0.268 | 0.123 | 2.97E-02 | 2.79E-01 | 8 | 63 | NA | NA |
| **acetaminophen** | **PE 16:0/18:1** | **0.249** | **0.074** | **7.76E-04** | **2.27E-02** | **7** | **57** | **NA** | **NA** |
| acetaminophen | Hydantoin-5-propionic acid | 0.234 | 0.117 | 4.51E-02 | 3.32E-01 | 9 | 65 | NA | NA |
| acetaminophen | 4-Hydroxyphenyl acetate | 0.231 | 0.088 | 8.56E-03 | 1.30E-01 | 12 | 66 | NA | NA |
| **acetaminophen** | **Gulonic acid** | **0.231** | **0.068** | **7.31E-04** | **2.21E-02** | **10** | **61** | **NA** | **NA** |
| **acetaminophen** | **PE 16:0/18:2** | **0.217** | **0.055** | **8.11E-05** | **4.32E-03** | **7** | **38** | **NA** | **NA** |
| acetaminophen | MG 0:0/18:1(9Z)/0:0 | 0.216 | 0.096 | 2.47E-02 | 2.49E-01 | 8 | 61 | NA | NA |
| acetaminophen | Sphingadienine | 0.211 | 0.100 | 3.52E-02 | 2.96E-01 | 5 | 12 | NA | NA |
| acetaminophen | Heptenedioic acid | 0.204 | 0.103 | 4.85E-02 | 3.46E-01 | 7 | 48 | NA | NA |
| acetaminophen | N(6)-Methyllysine | 0.202 | 0.094 | 3.08E-02 | 2.79E-01 | 7 | 7 | NA | NA |
| acetaminophen | CAR 20:0 | 0.201 | 0.086 | 1.94E-02 | 2.22E-01 | 8 | 58 | NA | NA |
| acetaminophen | 6-Hydroxyindole sulfate | 0.200 | 0.080 | 1.27E-02 | 1.75E-01 | 9 | 43 | NA | NA |
| **acetaminophen** | **PE 16:0/20:4** | **0.199** | **0.058** | **5.33E-04** | **1.67E-02** | **7** | **52** | **NA** | **NA** |
| acetaminophen | CAR DC3:0;2Me | 0.197 | 0.073 | 6.80E-03 | 1.21E-01 | 5 | 27 | NA | NA |
| acetaminophen | PE 18:0/22:6 | 0.196 | 0.087 | 2.52E-02 | 2.50E-01 | 5 | 36 | NA | NA |
| **acetaminophen** | **PE 18:0/18:2** | **0.195** | **0.048** | **5.56E-05** | **3.36E-03** | **7** | **25** | **NA** | **NA** |
| **acetaminophen** | **PE 18:0/18:1** | **0.190** | **0.053** | **3.74E-04** | **1.47E-02** | **7** | **24** | **NA** | **NA** |
| acetaminophen | N-Acetylhistidine | 0.188 | 0.080 | 1.80E-02 | 2.09E-01 | 11 | 68 | NA | NA |
| **acetaminophen** | **N-Acetylphenylalanine** | **0.188** | **0.059** | **1.46E-03** | **3.52E-02** | **14** | **58** | **-0.235** | **0.142** |
| acetaminophen | 6-oxopiperidine-2-carboxylic acid | 0.188 | 0.078 | 1.62E-02 | 1.91E-01 | 10 | 61 | NA | NA |
| **acetaminophen** | **Methylimidazoleacetic acid** | **0.185** | **0.044** | **2.60E-05** | **1.96E-03** | **10** | **29** | **NA** | **NA** |
| **acetaminophen** | **PE 18:0/20:4** | **0.185** | **0.049** | **1.86E-04** | **9.37E-03** | **7** | **46** | **NA** | **NA** |
| acetaminophen | 7Z,10Z-Hexadecadienoic acid | 0.180 | 0.090 | 4.58E-02 | 3.32E-01 | 8 | 29 | NA | NA |
| acetaminophen | delta-Valerobetaine | 0.177 | 0.083 | 3.41E-02 | 2.91E-01 | 7 | 50 | NA | NA |
| acetaminophen | Orotidine | 0.177 | 0.080 | 2.78E-02 | 2.70E-01 | 9 | 39 | NA | NA |
| **acetaminophen** | **N-Acetylhexosamine** | **0.171** | **0.046** | **1.98E-04** | **9.41E-03** | **10** | **66** | **NA** | **NA** |
| **acetaminophen** | **Methionine sulfone** | **0.171** | **0.050** | **5.36E-04** | **1.67E-02** | **9** | **39** | **NA** | **NA** |
| acetaminophen | N-Acetylglutamine | 0.170 | 0.071 | 1.61E-02 | 1.91E-01 | 10 | 58 | NA | NA |
| acetaminophen | Retinol | 0.166 | 0.063 | 8.54E-03 | 1.30E-01 | 11 | 78 | NA | NA |
| acetaminophen | Quinolinic acid | 0.164 | 0.075 | 2.88E-02 | 2.77E-01 | 10 | 56 | NA | NA |
| acetaminophen | alpha-N-Phenylacetylglutamine | 0.164 | 0.064 | 1.01E-02 | 1.50E-01 | 14 | 36 | -0.219 | 0.328 |
| **acetaminophen** | **LPE 16:0/0:0** | **0.162** | **0.051** | **1.48E-03** | **3.52E-02** | **8** | **63** | **NA** | **NA** |
| **acetaminophen** | **N-Acetyltaurine** | **0.159** | **0.049** | **1.26E-03** | **3.26E-02** | **9** | **47** | **NA** | **NA** |
| acetaminophen | 10Z-Heptadecenoic acid | 0.153 | 0.071 | 3.17E-02 | 2.79E-01 | 10 | 32 | NA | NA |
| acetaminophen | PI 16:0/20:4 | 0.151 | 0.073 | 3.90E-02 | 3.12E-01 | 6 | 59 | NA | NA |
| acetaminophen | LPE 0:0/18:0 | 0.149 | 0.059 | 1.19E-02 | 1.71E-01 | 7 | 54 | NA | NA |
| acetaminophen | N-Acetylneuraminic acid | 0.143 | 0.063 | 2.24E-02 | 2.42E-01 | 14 | 78 | -0.262 | 0.117 |
| **acetaminophen** | **LPE 18:0/0:0** | **0.139** | **0.040** | **5.04E-04** | **1.67E-02** | **10** | **46** | **NA** | **NA** |
| acetaminophen | 3-Hydroxymethylglutaric acid | 0.138 | 0.050 | 5.61E-03 | 1.04E-01 | 11 | 50 | NA | NA |
| acetaminophen | CAR 20:1 | 0.137 | 0.063 | 3.02E-02 | 2.79E-01 | 8 | 29 | NA | NA |
| acetaminophen | PI 16:0/18:2 | 0.135 | 0.059 | 2.15E-02 | 2.34E-01 | 7 | 51 | NA | NA |
| acetaminophen | 1-Methylinosine | 0.134 | 0.066 | 4.20E-02 | 3.22E-01 | 9 | 65 | NA | NA |
| **acetaminophen** | **Cer 18:0;O2/16:0** | **0.130** | **0.039** | **8.32E-04** | **2.35E-02** | **7** | **2** | **NA** | **NA** |
| acetaminophen | 5'-Methylthioadenosine | 0.126 | 0.052 | 1.47E-02 | 1.80E-01 | 15 | 46 | 0.115 | 0.518 |
| **acetaminophen** | **N-Acetylvaline** | **0.122** | **0.031** | **6.77E-05** | **3.83E-03** | **10** | **49** | **NA** | **NA** |
| acetaminophen | Arabinonic acid/Xylonic acid | 0.122 | 0.056 | 3.05E-02 | 2.79E-01 | 9 | 46 | NA | NA |
| acetaminophen | CAR 14:0 | 0.121 | 0.053 | 2.33E-02 | 2.42E-01 | 14 | 53 | NA | NA |
| acetaminophen | N6-Carbamoyl-L-threonyladenosine | 0.114 | 0.041 | 5.09E-03 | 1.00E-01 | 9 | 53 | NA | NA |
| acetaminophen | N-Acetylglutamic acid | 0.103 | 0.049 | 3.40E-02 | 2.91E-01 | 12 | 53 | NA | NA |
| acetaminophen | Methylsuccinic acid | 0.103 | 0.042 | 1.44E-02 | 1.78E-01 | 9 | 31 | NA | NA |
| acetaminophen | Homocitric acid | 0.102 | 0.047 | 3.13E-02 | 2.79E-01 | 7 | 48 | NA | NA |
| acetaminophen | Erythronic acid | 0.099 | 0.036 | 5.47E-03 | 1.04E-01 | 10 | 44 | NA | NA |
| acetaminophen | N6-Acetyllysine | 0.092 | 0.041 | 2.48E-02 | 2.49E-01 | 13 | 79 | NA | NA |
| acetaminophen | LPE 20:4/0:0 | 0.090 | 0.039 | 2.28E-02 | 2.42E-01 | 7 | 55 | NA | NA |
| acetaminophen | LPI 18:2/0:0 | 0.089 | 0.043 | 4.01E-02 | 3.15E-01 | 7 | 24 | NA | NA |
| acetaminophen | PI 18:0/18:2 | 0.084 | 0.034 | 1.33E-02 | 1.75E-01 | 7 | 17 | NA | NA |
| acetaminophen | CAR 16:0 | 0.081 | 0.040 | 4.58E-02 | 3.32E-01 | 16 | 55 | 0.205 | 0.157 |
| acetaminophen | PC 18:0/18:1 | 0.079 | 0.036 | 2.63E-02 | 2.59E-01 | 7 | 33 | NA | NA |
| acetaminophen | PC 16:0/18:1 | 0.079 | 0.030 | 8.33E-03 | 1.30E-01 | 7 | 56 | NA | NA |
| acetaminophen | Myo-inositol | 0.071 | 0.035 | 4.49E-02 | 3.32E-01 | 12 | 20 | NA | NA |
| acetaminophen | N-Acetylputrescine | 0.064 | 0.031 | 3.54E-02 | 2.96E-01 | 14 | 36 | NA | NA |
| acetaminophen | PC 16:0/18:0 | 0.056 | 0.026 | 3.37E-02 | 2.91E-01 | 7 | 36 | NA | NA |
| acetaminophen | PC 16:0/18:2 | 0.038 | 0.018 | 3.80E-02 | 3.07E-01 | 7 | 40 | NA | NA |
| acetaminophen | 3-Dehydroxycarnitine | -0.074 | 0.028 | 7.75E-03 | 1.28E-01 | 14 | 46 | NA | NA |
| acetaminophen | Ureidopropionic acid | -0.081 | 0.040 | 4.23E-02 | 3.22E-01 | 14 | 51 | NA | NA |
| acetaminophen | 2-Hydroxyisocaproic acid | -0.124 | 0.050 | 1.29E-02 | 1.75E-01 | 10 | 47 | NA | NA |
| acetaminophen | Eicosanedioic acid | -0.124 | 0.059 | 3.63E-02 | 3.02E-01 | 9 | 47 | NA | NA |
| acetaminophen | alpha-CEHC sulfate | -0.139 | 0.058 | 1.63E-02 | 1.91E-01 | 7 | 6 | NA | NA |
| acetaminophen | Homoarginine | -0.139 | 0.060 | 2.07E-02 | 2.34E-01 | 11 | 70 | NA | NA |
| acetaminophen | Docosanedioic acid | -0.145 | 0.074 | 4.80E-02 | 3.45E-01 | 6 | 47 | NA | NA |
| acetaminophen | Cryptoxanthin | -0.219 | 0.095 | 2.11E-02 | 2.34E-01 | 8 | 21 | NA | NA |
| acetaminophen | Sulfoglycolithocholic acid | -0.228 | 0.114 | 4.52E-02 | 3.32E-01 | 9 | 47 | NA | NA |
| acetaminophen | Deoxyuridine | -0.242 | 0.113 | 3.18E-02 | 2.79E-01 | 12 | 87 | 0.244 | 0.465 |
| acetaminophen | 5alpha-Androstane-3alpha-ol-17-one sulfate | -0.254 | 0.126 | 4.32E-02 | 3.26E-01 | 11 | 69 | NA | NA |
| acetaminophen | Vanillyl alcohol 4-sulfate | -0.265 | 0.100 | 7.93E-03 | 1.28E-01 | 8 | 61 | NA | NA |
| **acetaminophen** | **Ribothymidine** | **-0.277** | **0.064** | **1.62E-05** | **1.62E-03** | **12** | **93** | **NA** | **NA** |
| acetaminophen | 3-Indolepropionic acid | -0.319 | 0.112 | 4.39E-03 | 9.03E-02 | 13 | 47 | -0.321 | 0.336 |
| acetaminophen | Pregnenolone sulfate | -0.333 | 0.123 | 6.60E-03 | 1.19E-01 | 11 | 71 | NA | NA |
| acetaminophen | Taurolithocholic acid sulfate | -0.340 | 0.137 | 1.30E-02 | 1.75E-01 | 9 | 54 | NA | NA |
| acetaminophen | 17alpha-hydroxypregnenolone 3-sulfate | -0.346 | 0.140 | 1.37E-02 | 1.75E-01 | 5 | 74 | NA | NA |
| **acetaminophen** | **androstenediol (3beta_17beta) monosulfate (2)** | **-0.358** | **0.103** | **4.95E-04** | **1.67E-02** | **6** | **38** | **NA** | **NA** |
| acetaminophen | Dehydroepiandrosterone sulfate | -0.370 | 0.150 | 1.36E-02 | 1.75E-01 | 13 | 81 | 0.302 | 0.467 |
| acetaminophen | 3,7-Dimethyluric acid | -0.391 | 0.149 | 8.60E-03 | 1.30E-01 | 8 | 76 | NA | NA |
| **acetaminophen** | **5alpha-pregnan-3beta_20beta-diol monosulfate (1)** | **-0.420** | **0.104** | **5.50E-05** | **3.36E-03** | **7** | **46** | **NA** | **NA** |
| **acetaminophen** | **androstenediol (3alpha_ 17alpha) monosulfate (2)** | **-0.424** | **0.078** | **5.54E-08** | **1.67E-05** | **6** | **33** | **NA** | **NA** |
| **acetaminophen** | **5alpha-Androstan-3alpha,17beta-diol disulfate** | **-0.444** | **0.123** | **2.89E-04** | **1.19E-02** | **8** | **67** | **NA** | **NA** |
| **acetaminophen** | **5alpha-androstan-3alpha_17alpha-diol monosulfate** | **-0.464** | **0.100** | **3.74E-06** | **6.58E-04** | **6** | **62** | **NA** | **NA** |
| acetaminophen | 4-Androsten-3beta,17beta-diol disulfate | -0.481 | 0.195 | 1.36E-02 | 1.75E-01 | 11 | 84 | NA | NA |
| acetaminophen | androstenediol (3alpha_ 17alpha) monosulfate (3) | -0.482 | 0.189 | 1.06E-02 | 1.55E-01 | 8 | 80 | NA | NA |
| acetaminophen | Epiandrosterone | -0.507 | 0.179 | 4.69E-03 | 9.44E-02 | 10 | 81 | NA | NA |
| **acetaminophen** | **Taurocholenate sulfate** | **-0.542** | **0.119** | **5.32E-06** | **6.74E-04** | **10** | **73** | **NA** | **NA** |
| **acetaminophen** | **3beta,16alpha-Dihydroxyandrostenone sulfate** | **-0.553** | **0.158** | **4.53E-04** | **1.64E-02** | **6** | **63** | **NA** | **NA** |
| **acetaminophen** | **Glycocholenate sulfate** | **-0.621** | **0.105** | **3.77E-09** | **1.70E-06** | **10** | **84** | **NA** | **NA** |
| acetaminophen | 4-Allylcatechol sulfate | -0.621 | 0.287 | 3.07E-02 | 2.79E-01 | 5 | 61 | NA | NA |
| **acetaminophen** | **21-Hydroxypregnenolone** | **-0.711** | **0.167** | **2.10E-05** | **1.73E-03** | **10** | **86** | **NA** | **NA** |
| **acetaminophen** | **5alpha-Androstan-3beta,17alpha-diol disulfate** | **-0.714** | **0.155** | **4.36E-06** | **6.58E-04** | **8** | **79** | **NA** | **NA** |
| acetaminophen | pregnenetriol disulfate* | -0.721 | 0.288 | 1.24E-02 | 1.75E-01 | 5 | 86 | NA | NA |
| acetaminophen | pregnenediol disulfate (C21H34O8S2)* | -0.823 | 0.309 | 7.72E-03 | 1.28E-01 | 5 | 84 | NA | NA |
| **acetaminophen** | **5alpha-Androstane-3beta,17beta-diol disulfate** | **-0.910** | **0.290** | **1.70E-03** | **3.94E-02** | **9** | **89** | **NA** | **NA** |
| **acetaminophen** | **5alpha-Pregnan-3beta,20alpha-diol disulfate** | **-1.110** | **0.260** | **1.95E-05** | **1.73E-03** | **10** | **90** | **NA** | **NA** |

**Supplementary Table 3. Differential metabolites found by meta-analysis to be associated with metoprolol intake.** Bold font represents metabolites remaining significantly associated (p<0.05) after FDR correction. MS instrument was not included as a moderator in meta-analysis as there were less than 3 QTOF studies in each case.

| drug | metabolite | LogFC | SE | p_val | p.adj | I2 | number of studies |
| --- | --- | --- | --- | --- | --- | --- | --- |
| metoprolol | Salicylic acid | 1.243 | 0.535 | 2.01E-02 | 1.14E-01 | 89 | 6 |
| **metoprolol** | **Salicyluric acid** | **1.231** | **0.433** | **4.47E-03** | **4.39E-02** | **84** | **7** |
| metoprolol | 2-Furoylglycine | 0.610 | 0.224 | 6.48E-03 | 5.36E-02 | 81 | 6 |
| **metoprolol** | **Sucrose** | **0.498** | **0.162** | **2.06E-03** | **3.08E-02** | **42** | **6** |
| **metoprolol** | **P-Cresol glucuronide** | **0.476** | **0.150** | **1.53E-03** | **2.91E-02** | **21** | **6** |
| metoprolol | Indoleacetyl glutamine | 0.451 | 0.226 | 4.60E-02 | 1.81E-01 | 88 | 6 |
| metoprolol | 2-Hydroxyphenylacetic acid | 0.450 | 0.166 | 6.62E-03 | 5.41E-02 | 78 | 6 |
| **metoprolol** | **gamma-CEHC glucuronide** | **0.445** | **0.160** | **5.40E-03** | **4.70E-02** | **68** | **6** |
| metoprolol | 3-Hydroxyhippuric acid | 0.391 | 0.159 | 1.37E-02 | 9.05E-02 | 36 | 6 |
| **metoprolol** | **Glucuronic acid** | **0.384** | **0.107** | **3.14E-04** | **1.24E-02** | **65** | **6** |
| **metoprolol** | **3E-Methylglutaconic acid** | **0.375** | **0.104** | **2.94E-04** | **1.23E-02** | **68** | **6** |
| metoprolol | N-Acetylcitrulline | 0.367 | 0.144 | 1.06E-02 | 7.74E-02 | 73 | 7 |
| **metoprolol** | **Orotidine** | **0.365** | **0.131** | **5.40E-03** | **4.70E-02** | **57** | **6** |
| **metoprolol** | **CAR DC5:0;3Me** | **0.363** | **0.113** | **1.27E-03** | **2.75E-02** | **46** | **6** |
| metoprolol | Ectoine | 0.356 | 0.174 | 4.09E-02 | 1.68E-01 | 73 | 8 |
| metoprolol | 3-Methoxytyrosine | 0.330 | 0.143 | 2.11E-02 | 1.16E-01 | 91 | 7 |
| metoprolol | N-Acetylglutamine | 0.305 | 0.133 | 2.18E-02 | 1.19E-01 | 81 | 6 |
| **metoprolol** | **3-Hydroxymethylglutaric acid** | **0.301** | **0.086** | **4.96E-04** | **1.36E-02** | **73** | **6** |
| **metoprolol** | **alpha-N-Phenylacetylglutamine** | **0.300** | **0.099** | **2.54E-03** | **3.40E-02** | **54** | **7** |
| **metoprolol** | **1-Methylinosine** | **0.295** | **0.070** | **2.28E-05** | **2.45E-03** | **48** | **6** |
| metoprolol | 4-Hydroxyhippuric acid | 0.286 | 0.118 | 1.53E-02 | 9.73E-02 | 52 | 7 |
| metoprolol | Indolin-2-one | 0.276 | 0.107 | 1.01E-02 | 7.51E-02 | 54 | 7 |
| **metoprolol** | **Imidazole propanoate** | **0.273** | **0.088** | **1.94E-03** | **3.08E-02** | **31** | **7** |
| metoprolol | Vanillylmandelic acid | 0.269 | 0.106 | 1.07E-02 | 7.74E-02 | 79 | 6 |
| **metoprolol** | **Quinolinic acid** | **0.267** | **0.078** | **6.51E-04** | **1.64E-02** | **41** | **6** |
| metoprolol | Indoxyl sulfate | 0.260 | 0.099 | 8.26E-03 | 6.34E-02 | 52 | 6 |
| **metoprolol** | **N2,N2-Dimethylguanosine** | **0.260** | **0.078** | **9.09E-04** | **2.12E-02** | **81** | **7** |
| metoprolol | Acisoga | 0.258 | 0.120 | 3.14E-02 | 1.44E-01 | 73 | 6 |
| **metoprolol** | **N1-Methyl-2-pyridone-5-carboxamide** | **0.255** | **0.082** | **1.95E-03** | **3.08E-02** | **47** | **8** |
| metoprolol | N-Acetylphenylalanine | 0.255 | 0.108 | 1.88E-02 | 1.09E-01 | 82 | 6 |
| metoprolol | Cystathionine | 0.251 | 0.115 | 2.83E-02 | 1.37E-01 | 42 | 7 |
| metoprolol | 2-Aminobenzoic acid | 0.249 | 0.100 | 1.31E-02 | 8.79E-02 | 50 | 7 |
| metoprolol | 6-Hydroxyindole sulfate | 0.245 | 0.115 | 3.39E-02 | 1.53E-01 | 60 | 6 |
| metoprolol | Phenyl hydrogen sulfate | 0.241 | 0.107 | 2.37E-02 | 1.28E-01 | 40 | 6 |
| **metoprolol** | **4-Acetamidobutanoic acid** | **0.239** | **0.083** | **3.96E-03** | **4.36E-02** | **70** | **8** |
| metoprolol | Phenyl acetate | 0.235 | 0.119 | 4.82E-02 | 1.85E-01 | 42 | 5 |
| metoprolol | N4-Acetylcytidine | 0.235 | 0.095 | 1.38E-02 | 9.05E-02 | 61 | 8 |
| metoprolol | Isobutyrylglycine | 0.233 | 0.106 | 2.77E-02 | 1.36E-01 | 52 | 6 |
| **metoprolol** | **N6-Carbamoyl-L-threonyladenosine** | **0.225** | **0.076** | **3.32E-03** | **3.94E-02** | **76** | **6** |
| **metoprolol** | **CAR DC4:0** | **0.215** | **0.053** | **5.35E-05** | **4.81E-03** | **40** | **6** |
| metoprolol | 4,5-Dihydroorotic acid | 0.215 | 0.095 | 2.41E-02 | 1.28E-01 | 52 | 6 |
| metoprolol | N-Acetyltyrosine | 0.206 | 0.094 | 2.80E-02 | 1.37E-01 | 64 | 6 |
| metoprolol | S-Adenosylhomocysteine | 0.194 | 0.077 | 1.11E-02 | 7.77E-02 | 65 | 6 |
| metoprolol | Kynurenic acid | 0.193 | 0.097 | 4.60E-02 | 1.81E-01 | 76 | 7 |
| metoprolol | CAR 3:0;2Me | 0.189 | 0.079 | 1.66E-02 | 1.00E-01 | 46 | 7 |
| **metoprolol** | **Methylimidazoleacetic acid** | **0.189** | **0.063** | **2.74E-03** | **3.45E-02** | **44** | **7** |
| metoprolol | Arabinonic acid/Xylonic acid | 0.188 | 0.071 | 8.02E-03 | 6.32E-02 | 49 | 6 |
| **metoprolol** | **N-Acetylputrescine** | **0.187** | **0.036** | **1.63E-07** | **1.03E-04** | **20** | **7** |
| **metoprolol** | **N-Acetylserine** | **0.186** | **0.055** | **7.11E-04** | **1.72E-02** | **65** | **6** |
| metoprolol | Erythritol | 0.183 | 0.092 | 4.57E-02 | 1.81E-01 | 46 | 6 |
| **metoprolol** | **N-Acetylthreonine** | **0.177** | **0.058** | **2.20E-03** | **3.08E-02** | **58** | **6** |
| **metoprolol** | **N-Acetylglutamic acid** | **0.175** | **0.062** | **4.41E-03** | **4.39E-02** | **36** | **6** |
| metoprolol | LPG 16:0/0:0 | 0.170 | 0.075 | 2.39E-02 | 1.28E-01 | 43 | 5 |
| metoprolol | p-Cresol sulfate | 0.169 | 0.085 | 4.73E-02 | 1.82E-01 | 0 | 6 |
| metoprolol | C-Glycosyltryptophan | 0.166 | 0.068 | 1.46E-02 | 9.49E-02 | 75 | 6 |
| metoprolol | PE 16:0/20:4 | 0.165 | 0.083 | 4.67E-02 | 1.82E-01 | 68 | 5 |
| **metoprolol** | **Arabitol** | **0.162** | **0.058** | **5.46E-03** | **4.70E-02** | **59** | **6** |
| metoprolol | 1-Methylhistidine | 0.157 | 0.062 | 1.13E-02 | 7.78E-02 | 40 | 7 |
| metoprolol | 5'-Methylthioadenosine | 0.156 | 0.071 | 2.72E-02 | 1.36E-01 | 64 | 7 |
| **metoprolol** | **N-Acetylneuraminic acid** | **0.152** | **0.054** | **4.81E-03** | **4.59E-02** | **57** | **6** |
| **metoprolol** | **N-Acetyltaurine** | **0.152** | **0.048** | **1.44E-03** | **2.91E-02** | **17** | **6** |
| **metoprolol** | **2-Oxoglutaric acid** | **0.150** | **0.054** | **5.12E-03** | **4.67E-02** | **25** | **6** |
| metoprolol | Erythronic acid | 0.147 | 0.071 | 4.01E-02 | 1.67E-01 | 76 | 6 |
| metoprolol | Picolinic acid | 0.145 | 0.061 | 1.74E-02 | 1.03E-01 | 3 | 6 |
| metoprolol | Sulfotyrosine | 0.141 | 0.055 | 1.08E-02 | 7.74E-02 | 52 | 6 |
| metoprolol | CAR 4:0;2Me | 0.139 | 0.066 | 3.67E-02 | 1.59E-01 | 25 | 6 |
| **metoprolol** | **N-Acetylmethionine** | **0.137** | **0.048** | **4.27E-03** | **4.39E-02** | **19** | **7** |
| **metoprolol** | **N-Formylmethionine** | **0.136** | **0.038** | **3.53E-04** | **1.24E-02** | **45** | **6** |
| metoprolol | Myo-inositol | 0.135 | 0.062 | 2.89E-02 | 1.38E-01 | 41 | 6 |
| metoprolol | Urea | 0.131 | 0.056 | 1.91E-02 | 1.09E-01 | 49 | 6 |
| metoprolol | Pseudouridine | 0.129 | 0.049 | 8.04E-03 | 6.32E-02 | 68 | 7 |
| metoprolol | 2-Hydroxyglutaric acid | 0.125 | 0.054 | 2.05E-02 | 1.15E-01 | 37 | 6 |
| **metoprolol** | **N-Acetylvaline** | **0.125** | **0.043** | **3.79E-03** | **4.33E-02** | **62** | **6** |
| metoprolol | gamma-Glutamylglutamic acid | 0.122 | 0.047 | 8.98E-03 | 6.80E-02 | 7 | 6 |
| metoprolol | Cystine | 0.121 | 0.058 | 3.69E-02 | 1.59E-01 | 34 | 7 |
| metoprolol | 4-Hydroxyphenylpyruvic acid | 0.118 | 0.054 | 2.93E-02 | 1.38E-01 | 28 | 6 |
| **metoprolol** | **N-Acetylhexosamine** | **0.117** | **0.040** | **3.60E-03** | **4.20E-02** | **30** | **6** |
| **metoprolol** | **N6-Acetyllysine** | **0.115** | **0.037** | **2.07E-03** | **3.08E-02** | **53** | **8** |
| metoprolol | gamma-Glutamylvaline | 0.114 | 0.043 | 7.68E-03 | 6.19E-02 | 22 | 6 |
| **metoprolol** | **1-Methyladenosine** | **0.109** | **0.036** | **2.20E-03** | **3.08E-02** | **65** | **8** |
| **metoprolol** | **gamma-Glutamylleucine** | **0.108** | **0.035** | **2.08E-03** | **3.08E-02** | **8** | **7** |
| **metoprolol** | **gamma-Glutamylphenylalanine** | **0.107** | **0.035** | **2.11E-03** | **3.08E-02** | **8** | **6** |
| metoprolol | N-6-Trimethyllysine | 0.097 | 0.048 | 4.43E-02 | 1.78E-01 | 35 | 8 |
| metoprolol | gamma-Glutamylisoleucine | 0.093 | 0.042 | 2.69E-02 | 1.35E-01 | 15 | 6 |
| metoprolol | N-Acetylalanine | 0.088 | 0.044 | 4.36E-02 | 1.77E-01 | 74 | 7 |
| metoprolol | Creatinine | 0.083 | 0.034 | 1.29E-02 | 8.74E-02 | 58 | 8 |
| metoprolol | 7-Methylguanine | 0.078 | 0.035 | 2.49E-02 | 1.30E-01 | 55 | 7 |
| metoprolol | Sulfate | 0.070 | 0.035 | 4.92E-02 | 1.86E-01 | 47 | 6 |
| metoprolol | Dimethylarginine | 0.068 | 0.032 | 3.53E-02 | 1.55E-01 | 56 | 6 |
| metoprolol | Proline | 0.067 | 0.032 | 3.46E-02 | 1.53E-01 | 39 | 8 |
| metoprolol | PC 16:0/18:2 | -0.041 | 0.019 | 3.46E-02 | 1.53E-01 | 31 | 5 |
| metoprolol | PC 18:0/18:2 | -0.060 | 0.022 | 6.44E-03 | 5.36E-02 | 30 | 5 |
| metoprolol | PC 16:0/18:0 | -0.062 | 0.030 | 3.79E-02 | 1.62E-01 | 30 | 5 |
| metoprolol | SM 42:3;O2 | -0.064 | 0.030 | 3.64E-02 | 1.59E-01 | 22 | 5 |
| metoprolol | PC 18:0/18:1 | -0.065 | 0.033 | 4.57E-02 | 1.81E-01 | 0 | 5 |
| metoprolol | SM 38:1;O2 | -0.069 | 0.028 | 1.26E-02 | 8.59E-02 | 18 | 5 |
| metoprolol | Uridine | -0.072 | 0.035 | 4.31E-02 | 1.76E-01 | 30 | 7 |
| metoprolol | SM 42:2;O2 | -0.080 | 0.040 | 4.68E-02 | 1.82E-01 | 43 | 5 |
| metoprolol | LPC 18:1/0:0 | -0.082 | 0.039 | 3.38E-02 | 1.53E-01 | 32 | 6 |
| metoprolol | LPC 18:2/0:0 | -0.083 | 0.037 | 2.57E-02 | 1.32E-01 | 6 | 5 |
| metoprolol | CAR 18:0 | -0.085 | 0.039 | 2.94E-02 | 1.38E-01 | 15 | 7 |
| **metoprolol** | **SM 32:1;O2** | **-0.090** | **0.031** | **4.35E-03** | **4.39E-02** | **17** | **5** |
| metoprolol | Arachidic acid | -0.094 | 0.041 | 2.11E-02 | 1.16E-01 | 33 | 6 |
| **metoprolol** | **sn-Glycero-3-phosphoethanolamine** | **-0.094** | **0.032** | **2.96E-03** | **3.65E-02** | **23** | **6** |
| metoprolol | SM 40:2;O2 | -0.095 | 0.043 | 2.69E-02 | 1.35E-01 | 46 | 5 |
| metoprolol | SM 18:1;O2/22:0 | -0.096 | 0.040 | 1.58E-02 | 9.82E-02 | 47 | 5 |
| metoprolol | LacCer 18:1;O2/16:0 | -0.096 | 0.040 | 1.62E-02 | 9.91E-02 | 54 | 5 |
| metoprolol | CAR 24:0 | -0.098 | 0.048 | 4.01E-02 | 1.67E-01 | 2 | 5 |
| metoprolol | PC P-16:0/18:1 or PC O-16:1/18:1 | -0.100 | 0.041 | 1.51E-02 | 9.69E-02 | 41 | 5 |
| metoprolol | PE P-18:0/18:2 or PE O-18:1/18:2 | -0.104 | 0.043 | 1.56E-02 | 9.80E-02 | 13 | 5 |
| **metoprolol** | **SM 35:1;O2** | **-0.107** | **0.033** | **1.31E-03** | **2.75E-02** | **31** | **5** |
| metoprolol | PC P-16:0/18:2 or PC O-16:1/18:2 | -0.107 | 0.040 | 8.26E-03 | 6.34E-02 | 37 | 5 |
| **metoprolol** | **Cholesterol** | **-0.107** | **0.030** | **3.91E-04** | **1.24E-02** | **15** | **6** |
| metoprolol | 2-Aminobutyric acid | -0.114 | 0.054 | 3.43E-02 | 1.53E-01 | 52 | 6 |
| metoprolol | SM 32:2;O2 | -0.114 | 0.048 | 1.62E-02 | 9.91E-02 | 41 | 5 |
| metoprolol | CAR 20:0 | -0.118 | 0.058 | 4.10E-02 | 1.68E-01 | 22 | 5 |
| metoprolol | HexCer 18:1;O2/18:0 | -0.133 | 0.056 | 1.69E-02 | 1.01E-01 | 48 | 5 |
| **metoprolol** | **sn-Glycero-3-phosphocholine** | **-0.133** | **0.045** | **3.27E-03** | **3.94E-02** | **38** | **8** |
| **metoprolol** | **CAR 14:0** | **-0.134** | **0.047** | **4.37E-03** | **4.39E-02** | **10** | **7** |
| metoprolol | Methylcysteine | -0.138 | 0.054 | 1.11E-02 | 7.77E-02 | 18 | 6 |
| **metoprolol** | **HexCer 18:1;O2/16:0** | **-0.142** | **0.047** | **2.41E-03** | **3.29E-02** | **51** | **5** |
| metoprolol | LPI 18:0/0:0 | -0.146 | 0.062 | 1.87E-02 | 1.09E-01 | 39 | 5 |
| **metoprolol** | **Capric acid** | **-0.146** | **0.052** | **4.94E-03** | **4.64E-02** | **3** | **6** |
| **metoprolol** | **Pentadecylic acid** | **-0.152** | **0.043** | **4.14E-04** | **1.24E-02** | **25** | **6** |
| **metoprolol** | **SM 41:2;O2** | **-0.152** | **0.040** | **1.62E-04** | **9.04E-03** | **29** | **5** |
| **metoprolol** | **PC 18:2/18:2** | **-0.153** | **0.050** | **2.06E-03** | **3.08E-02** | **26** | **5** |
| metoprolol | Adrenic acid | -0.155 | 0.072 | 3.13E-02 | 1.44E-01 | 30 | 6 |
| **metoprolol** | **Stearic acid** | **-0.155** | **0.041** | **1.52E-04** | **9.04E-03** | **32** | **7** |
| metoprolol | CAR 12:0 | -0.156 | 0.071 | 2.89E-02 | 1.38E-01 | 39 | 8 |
| metoprolol | Dodecanedioic acid | -0.156 | 0.069 | 2.44E-02 | 1.29E-01 | 13 | 6 |
| metoprolol | CAR 22:0 | -0.156 | 0.070 | 2.55E-02 | 1.32E-01 | 7 | 5 |
| **metoprolol** | **SM 39:1;O2** | **-0.162** | **0.046** | **4.57E-04** | **1.31E-02** | **29** | **5** |
| metoprolol | LPI 18:1/0:0 | -0.165 | 0.080 | 4.00E-02 | 1.67E-01 | 41 | 5 |
| **metoprolol** | **CAR 20:1** | **-0.168** | **0.060** | **5.20E-03** | **4.67E-02** | **9** | **5** |
| **metoprolol** | **Palmitic acid** | **-0.173** | **0.046** | **2.01E-04** | **9.04E-03** | **9** | **6** |
| metoprolol | 3-Hydroxydecanoic acid | -0.178 | 0.083 | 3.13E-02 | 1.44E-01 | 52 | 6 |
| **metoprolol** | **Dihomo-gamma-linolenic acid** | **-0.179** | **0.059** | **2.60E-03** | **3.41E-02** | **24** | **6** |
| **metoprolol** | **CAR 16:1** | **-0.180** | **0.063** | **4.20E-03** | **4.39E-02** | **38** | **6** |
| metoprolol | Lauric acid | -0.184 | 0.071 | 9.51E-03 | 7.12E-02 | 20 | 6 |
| **metoprolol** | **cis-Erucic acid** | **-0.193** | **0.069** | **5.11E-03** | **4.67E-02** | **29** | **6** |
| **metoprolol** | **CAR 14:1** | **-0.198** | **0.069** | **3.89E-03** | **4.36E-02** | **26** | **8** |
| **metoprolol** | **13Z,16Z-Docosadienoic acid** | **-0.202** | **0.054** | **1.81E-04** | **9.04E-03** | **6** | **6** |
| metoprolol | PC 16:1_18:3 | -0.202 | 0.092 | 2.76E-02 | 1.36E-01 | 47 | 5 |
| metoprolol | PC 18:2/18:3 | -0.208 | 0.093 | 2.47E-02 | 1.29E-01 | 55 | 5 |
| **metoprolol** | **DHA** | **-0.215** | **0.069** | **1.92E-03** | **3.08E-02** | **15** | **6** |
| **metoprolol** | **Tetradecanedioic acid** | **-0.219** | **0.069** | **1.51E-03** | **2.91E-02** | **8** | **6** |
| metoprolol | Myristoleic acid | -0.221 | 0.093 | 1.79E-02 | 1.05E-01 | 49 | 7 |
| metoprolol | Perfluorooctanesulfonic acid | -0.228 | 0.111 | 3.88E-02 | 1.65E-01 | 35 | 5 |
| **metoprolol** | **Linoleic acid** | **-0.231** | **0.065** | **4.01E-04** | **1.24E-02** | **15** | **6** |
| metoprolol | gamma-Glutamyl-2-aminobutanoate | -0.232 | 0.085 | 6.30E-03 | 5.36E-02 | 35 | 6 |
| **metoprolol** | **DPA** | **-0.234** | **0.072** | **1.10E-03** | **2.48E-02** | **6** | **6** |
| **metoprolol** | **Octadecatrienoic acid** | **-0.234** | **0.078** | **2.70E-03** | **3.45E-02** | **9** | **6** |
| **metoprolol** | **Margaric acid** | **-0.240** | **0.051** | **2.19E-06** | **4.58E-04** | **1** | **6** |
| **metoprolol** | **11Z,14Z-Eicosadienoic acid** | **-0.243** | **0.065** | **1.95E-04** | **9.04E-03** | **11** | **6** |
| **metoprolol** | **Carotene diol** | **-0.245** | **0.085** | **4.02E-03** | **4.36E-02** | **42** | **5** |
| metoprolol | Ergothioneine | -0.247 | 0.125 | 4.85E-02 | 1.85E-01 | 64 | 6 |
| **metoprolol** | **Myristic acid** | **-0.253** | **0.073** | **5.79E-04** | **1.52E-02** | **28** | **6** |
| **metoprolol** | **10-Hendecenoic acid** | **-0.258** | **0.084** | **2.03E-03** | **3.08E-02** | **55** | **6** |
| **metoprolol** | **Oleic acid** | **-0.261** | **0.062** | **2.34E-05** | **2.45E-03** | **11** | **6** |
| **metoprolol** | **5-Dodecenoic acid** | **-0.280** | **0.091** | **2.19E-03** | **3.08E-02** | **26** | **6** |
| **metoprolol** | **3-Hydroxydodecanoic acid** | **-0.289** | **0.072** | **6.62E-05** | **5.21E-03** | **17** | **6** |
| **metoprolol** | **Palmitoleic acid** | **-0.310** | **0.110** | **4.76E-03** | **4.59E-02** | **39** | **7** |
| **metoprolol** | **10Z-Heptadecenoic acid** | **-0.311** | **0.087** | **3.60E-04** | **1.24E-02** | **30** | **6** |
| **metoprolol** | **cis-Gondoic acid** | **-0.329** | **0.065** | **3.85E-07** | **1.21E-04** | **6** | **6** |
| **metoprolol** | **Nonadeca-10Z-enoic acid** | **-0.335** | **0.075** | **8.79E-06** | **1.38E-03** | **19** | **6** |
| **metoprolol** | **3-Hydroxybutyric acid** | **-0.389** | **0.102** | **1.30E-04** | **9.04E-03** | **18** | **7** |

**Supplementary Table 4. Differential metabolites found by meta-analysis to be associated with atenolol intake.** Bold font represents metabolites remaining significantly associated (p<0.05) after FDR correction. MS instrument was not included as a moderator in meta-analysis as there were less than 3 QTOF studies in each case.

| drug | metabolite | LogFC | SE | p_val | p.adj | I2 | number of studies |
| --- | --- | --- | --- | --- | --- | --- | --- |
| atenolol | Salicyluric acid | 1.066 | 0.382 | 5.29E-03 | 1.64E-01 | 67 | 6 |
| atenolol | Salicyluric glucuronide | 0.541 | 0.261 | 3.83E-02 | 2.85E-01 | 81 | 5 |
| atenolol | Homocitrulline | 0.444 | 0.143 | 1.95E-03 | 9.91E-02 | 64 | 6 |
| **atenolol** | **Glucuronic acid** | **0.430** | **0.109** | **7.93E-05** | **1.48E-02** | **37** | **5** |
| atenolol | CAR DC5:0;3Me | 0.425 | 0.168 | 1.15E-02 | 1.69E-01 | 57 | 5 |
| atenolol | Indole-3-methyl acetate | 0.419 | 0.166 | 1.18E-02 | 1.69E-01 | 52 | 5 |
| atenolol | N-Acetyl-3-methylhistidine | 0.415 | 0.173 | 1.64E-02 | 1.95E-01 | 55 | 5 |
| atenolol | Hexanoylglycine | 0.415 | 0.119 | 4.90E-04 | 5.47E-02 | 51 | 6 |
| atenolol | Isobutyrylglycine | 0.394 | 0.164 | 1.65E-02 | 1.95E-01 | 62 | 5 |
| atenolol | 3E-Methylglutaconic acid | 0.383 | 0.189 | 4.24E-02 | 2.88E-01 | 83 | 5 |
| atenolol | Gulonic acid | 0.378 | 0.175 | 3.02E-02 | 2.49E-01 | 68 | 5 |
| atenolol | Trigonelline | 0.377 | 0.183 | 3.98E-02 | 2.85E-01 | 51 | 6 |
| atenolol | 3-Hydroxymethylglutaric acid | 0.372 | 0.133 | 5.07E-03 | 1.64E-01 | 77 | 5 |
| atenolol | P-Cresol glucuronide | 0.365 | 0.179 | 4.13E-02 | 2.88E-01 | 4 | 5 |
| atenolol | gamma-CEHC glucuronide | 0.363 | 0.184 | 4.85E-02 | 2.97E-01 | 56 | 5 |
| atenolol | 1-Methyl nicotinamide | 0.357 | 0.149 | 1.67E-02 | 1.95E-01 | 65 | 6 |
| atenolol | Glycochenodeoxycholic acid 3-glucuronide | 0.355 | 0.149 | 1.69E-02 | 1.95E-01 | 19 | 5 |
| atenolol | Sorbitol | 0.352 | 0.131 | 7.02E-03 | 1.65E-01 | 11 | 5 |
| atenolol | Cystathionine | 0.337 | 0.149 | 2.40E-02 | 2.27E-01 | 41 | 6 |
| atenolol | Erythritol | 0.334 | 0.147 | 2.35E-02 | 2.26E-01 | 63 | 5 |
| atenolol | Myo-inositol | 0.333 | 0.132 | 1.16E-02 | 1.69E-01 | 75 | 5 |
| atenolol | Orotidine | 0.328 | 0.115 | 4.23E-03 | 1.57E-01 | 2 | 5 |
| atenolol | N1-Methyl-2-pyridone-5-carboxamide | 0.326 | 0.118 | 5.58E-03 | 1.64E-01 | 58 | 7 |
| atenolol | 2-Hydroxyphenylacetic acid | 0.323 | 0.162 | 4.68E-02 | 2.97E-01 | 59 | 5 |
| atenolol | CAR DC7:0 | 0.321 | 0.117 | 6.31E-03 | 1.65E-01 | 34 | 5 |
| atenolol | N-Acetylcitrulline | 0.319 | 0.141 | 2.33E-02 | 2.26E-01 | 54 | 6 |
| atenolol | Vanillylmandelic acid | 0.315 | 0.121 | 9.61E-03 | 1.65E-01 | 71 | 5 |
| **atenolol** | **1-Methylinosine** | **0.313** | **0.087** | **3.27E-04** | **4.56E-02** | **37** | **5** |
| atenolol | N2,N5-Diacetylornithine | 0.310 | 0.153 | 4.24E-02 | 2.88E-01 | 55 | 5 |
| atenolol | 4-Hydroxyhippuric acid | 0.267 | 0.102 | 8.87E-03 | 1.65E-01 | 3 | 6 |
| atenolol | Orotic acid | 0.264 | 0.084 | 1.60E-03 | 9.91E-02 | 8 | 5 |
| atenolol | Arabitol | 0.262 | 0.079 | 9.73E-04 | 8.40E-02 | 57 | 5 |
| atenolol | Pro-Gly | 0.256 | 0.118 | 2.97E-02 | 2.49E-01 | 24 | 5 |
| atenolol | N2,N2-Dimethylguanosine | 0.253 | 0.118 | 3.21E-02 | 2.52E-01 | 84 | 6 |
| atenolol | 4-Acetamidobutanoic acid | 0.251 | 0.096 | 8.97E-03 | 1.65E-01 | 67 | 7 |
| atenolol | Arabinonic acid/Xylonic acid | 0.241 | 0.109 | 2.69E-02 | 2.38E-01 | 57 | 5 |
| atenolol | 1-Methylhistidine | 0.238 | 0.094 | 1.15E-02 | 1.69E-01 | 57 | 6 |
| atenolol | Erythronic acid | 0.232 | 0.094 | 1.38E-02 | 1.83E-01 | 73 | 5 |
| atenolol | 4-Hydroxyphenyl acetate | 0.231 | 0.115 | 4.43E-02 | 2.94E-01 | 18 | 5 |
| atenolol | CAR 3:0;2Me | 0.227 | 0.089 | 1.12E-02 | 1.69E-01 | 35 | 6 |
| atenolol | N-Acetylthreonine | 0.219 | 0.075 | 3.65E-03 | 1.46E-01 | 52 | 5 |
| atenolol | N4-Acetylcytidine | 0.216 | 0.108 | 4.52E-02 | 2.94E-01 | 55 | 7 |
| atenolol | Phenylpyruvic acid | 0.211 | 0.103 | 3.95E-02 | 2.85E-01 | 65 | 6 |
| atenolol | Trimethylamine N-oxide | 0.210 | 0.096 | 2.93E-02 | 2.49E-01 | 26 | 7 |
| atenolol | PE 16:0/18:1 | 0.209 | 0.097 | 3.19E-02 | 2.52E-01 | 39 | 5 |
| atenolol | PE 16:0/20:4 | 0.203 | 0.088 | 2.03E-02 | 2.10E-01 | 47 | 5 |
| atenolol | PE 18:0/20:4 | 0.201 | 0.088 | 2.23E-02 | 2.22E-01 | 55 | 5 |
| atenolol | N-Acetylserine | 0.200 | 0.068 | 3.34E-03 | 1.43E-01 | 55 | 5 |
| atenolol | Methylimidazoleacetic acid | 0.198 | 0.083 | 1.64E-02 | 1.95E-01 | 42 | 6 |
| **atenolol** | **N6-Acetyllysine** | **0.198** | **0.049** | **5.17E-05** | **1.44E-02** | **57** | **7** |
| atenolol | Methionine sulfone | 0.195 | 0.061 | 1.36E-03 | 9.45E-02 | 1 | 5 |
| atenolol | N-Acetylneuraminic acid | 0.194 | 0.059 | 1.05E-03 | 8.40E-02 | 37 | 5 |
| atenolol | Kynurenic acid | 0.193 | 0.092 | 3.63E-02 | 2.81E-01 | 56 | 6 |
| atenolol | N(2)-Acetyllysine | 0.192 | 0.097 | 4.79E-02 | 2.97E-01 | 43 | 6 |
| atenolol | Quinolinic acid | 0.191 | 0.081 | 1.81E-02 | 1.95E-01 | 8 | 5 |
| atenolol | Glycocholenate sulfate | 0.188 | 0.073 | 9.75E-03 | 1.65E-01 | 1 | 5 |
| atenolol | 5'-Methylthioadenosine | 0.181 | 0.069 | 8.61E-03 | 1.65E-01 | 38 | 6 |
| atenolol | N6-Carbamoyl-L-threonyladenosine | 0.175 | 0.056 | 1.86E-03 | 9.91E-02 | 17 | 5 |
| atenolol | gamma-Glutamylisoleucine | 0.172 | 0.064 | 6.76E-03 | 1.65E-01 | 30 | 5 |
| atenolol | N-Acetyl-tryptophan | 0.172 | 0.085 | 4.42E-02 | 2.94E-01 | 47 | 6 |
| **atenolol** | **N-Acetylvaline** | **0.167** | **0.040** | **3.38E-05** | **1.44E-02** | **21** | **5** |
| atenolol | C-Glycosyltryptophan | 0.166 | 0.075 | 2.65E-02 | 2.38E-01 | 60 | 5 |
| atenolol | N-Acetylisoleucine | 0.162 | 0.066 | 1.45E-02 | 1.88E-01 | 4 | 5 |
| atenolol | Pseudouridine | 0.155 | 0.061 | 1.11E-02 | 1.69E-01 | 65 | 6 |
| atenolol | Pyruvic acid | 0.153 | 0.061 | 1.25E-02 | 1.75E-01 | 30 | 6 |
| atenolol | Sulfate | 0.152 | 0.050 | 2.16E-03 | 1.00E-01 | 51 | 5 |
| atenolol | Sulfotyrosine | 0.152 | 0.069 | 2.80E-02 | 2.44E-01 | 43 | 5 |
| atenolol | CAR DC4:0 | 0.149 | 0.075 | 4.75E-02 | 2.97E-01 | 46 | 5 |
| atenolol | 4-Hydroxyphenylpyruvic acid | 0.146 | 0.073 | 4.50E-02 | 2.94E-01 | 25 | 5 |
| atenolol | 3-Hydroxyisovaleric acid | 0.145 | 0.067 | 3.11E-02 | 2.52E-01 | 17 | 5 |
| atenolol | N-6-Trimethyllysine | 0.135 | 0.052 | 9.69E-03 | 1.65E-01 | 16 | 7 |
| atenolol | N-Formylmethionine | 0.135 | 0.057 | 1.76E-02 | 1.95E-01 | 53 | 5 |
| atenolol | gamma-Glutamylphenylalanine | 0.134 | 0.056 | 1.72E-02 | 1.95E-01 | 32 | 5 |
| atenolol | N-Acetylalanine | 0.126 | 0.048 | 8.29E-03 | 1.65E-01 | 62 | 6 |
| atenolol | Retinol | 0.119 | 0.046 | 9.42E-03 | 1.65E-01 | 12 | 6 |
| atenolol | cis-Aconitic acid | 0.115 | 0.051 | 2.53E-02 | 2.35E-01 | 34 | 5 |
| atenolol | 1-Methyladenosine | 0.110 | 0.041 | 7.91E-03 | 1.65E-01 | 55 | 7 |
| atenolol | Citrulline | 0.109 | 0.039 | 5.28E-03 | 1.64E-01 | 24 | 7 |
| atenolol | Kynurenine | 0.100 | 0.041 | 1.32E-02 | 1.80E-01 | 16 | 6 |
| atenolol | Creatinine | 0.084 | 0.037 | 2.16E-02 | 2.19E-01 | 43 | 7 |
| atenolol | 7-Methylguanine | 0.081 | 0.031 | 8.25E-03 | 1.65E-01 | 9 | 6 |
| atenolol | sn-Glycero-3-phosphoethanolamine | -0.081 | 0.041 | 4.80E-02 | 2.97E-01 | 18 | 5 |
| atenolol | HexCer 18:1;O2/16:0 | -0.109 | 0.046 | 1.79E-02 | 1.95E-01 | 6 | 5 |
| atenolol | LPE P-18:1 or LPE O-18:2 | -0.151 | 0.065 | 1.94E-02 | 2.05E-01 | 21 | 5 |
| atenolol | cis-Erucic acid | -0.164 | 0.080 | 3.99E-02 | 2.85E-01 | 6 | 5 |
| atenolol | 2-Hydroxycapric acid | -0.201 | 0.093 | 3.04E-02 | 2.49E-01 | 39 | 5 |
| atenolol | cis-Gondoic acid | -0.202 | 0.098 | 3.91E-02 | 2.85E-01 | 20 | 5 |
| atenolol | 3-Hydroxydecanoic acid | -0.222 | 0.113 | 4.96E-02 | 3.01E-01 | 54 | 5 |
| atenolol | Nonadeca-10Z-enoic acid | -0.223 | 0.107 | 3.71E-02 | 2.84E-01 | 24 | 5 |
| atenolol | Myristic acid | -0.225 | 0.101 | 2.63E-02 | 2.38E-01 | 30 | 5 |
| atenolol | 9,10-DiHOME | -0.258 | 0.097 | 7.81E-03 | 1.65E-01 | 3 | 5 |
| atenolol | Octadecatrienoic acid | -0.296 | 0.145 | 4.04E-02 | 2.85E-01 | 48 | 5 |

**Supplementary Table 5. Differential metabolites found by meta-analysis to be associated with hydrochlorothiazide intake.** Bold font represents metabolites remaining significantly associated (p<0.05) after FDR correction. MS instrument was not included as a moderator in meta-analysis as there were less than 3 QTOF studies in each case.

| drug | metabolite | LogFC | SE | p_val | p.adj | I2 | number of studies |
| --- | --- | --- | --- | --- | --- | --- | --- |
| hydrochlorothiazide | Taurodeoxycholic acid | 0.679 | 0.263 | 9.77E-03 | 1.38E-01 | 46 | 5 |
| hydrochlorothiazide | Stachydrine | 0.668 | 0.290 | 2.10E-02 | 1.78E-01 | 64 | 5 |
| hydrochlorothiazide | Taurolithocholic acid sulfate | 0.628 | 0.302 | 3.77E-02 | 2.23E-01 | 78 | 5 |
| hydrochlorothiazide | N-Acetyl-3-methylhistidine | 0.561 | 0.255 | 2.82E-02 | 1.99E-01 | 81 | 5 |
| hydrochlorothiazide | 1D-chiro-Inositol | 0.519 | 0.261 | 4.63E-02 | 2.44E-01 | 63 | 5 |
| hydrochlorothiazide | Lenticin | 0.479 | 0.208 | 2.10E-02 | 1.78E-01 | 36 | 5 |
| hydrochlorothiazide | gamma-CEHC glucuronide | 0.457 | 0.182 | 1.24E-02 | 1.51E-01 | 59 | 5 |
| hydrochlorothiazide | Theophylline | 0.453 | 0.197 | 2.12E-02 | 1.78E-01 | 20 | 5 |
| hydrochlorothiazide | Taurochenodeoxycholic acid | 0.430 | 0.155 | 5.60E-03 | 1.18E-01 | 8 | 5 |
| hydrochlorothiazide | N2,N5-Diacetylornithine | 0.428 | 0.188 | 2.29E-02 | 1.83E-01 | 74 | 5 |
| hydrochlorothiazide | Taurocholic acid | 0.405 | 0.175 | 2.08E-02 | 1.78E-01 | 6 | 5 |
| hydrochlorothiazide | Acisoga | 0.393 | 0.184 | 3.28E-02 | 2.12E-01 | 80 | 5 |
| **hydrochlorothiazide** | **Methionine sulfone** | **0.391** | **0.056** | **1.97E-12** | **8.62E-10** | **1** | **5** |
| hydrochlorothiazide | 3-Hydroxysebacic acid | 0.366 | 0.170 | 3.08E-02 | 2.11E-01 | 49 | 5 |
| hydrochlorothiazide | Maleic acid | 0.363 | 0.183 | 4.67E-02 | 2.44E-01 | 76 | 5 |
| hydrochlorothiazide | Dimethyl sulfone | 0.357 | 0.122 | 3.53E-03 | 1.18E-01 | 26 | 5 |
| hydrochlorothiazide | Orotidine | 0.350 | 0.151 | 2.03E-02 | 1.78E-01 | 50 | 5 |
| hydrochlorothiazide | N4-Acetylcytidine | 0.335 | 0.125 | 7.22E-03 | 1.18E-01 | 50 | 5 |
| hydrochlorothiazide | Homocitrulline | 0.330 | 0.100 | 9.98E-04 | 8.74E-02 | 32 | 5 |
| hydrochlorothiazide | CAR DC5:0;3Me | 0.327 | 0.145 | 2.38E-02 | 1.83E-01 | 49 | 5 |
| hydrochlorothiazide | CAR 4:0;3OH | 0.320 | 0.128 | 1.22E-02 | 1.51E-01 | 13 | 5 |
| hydrochlorothiazide | N-methyl pipecolic acid | 0.308 | 0.104 | 2.97E-03 | 1.18E-01 | 6 | 5 |
| hydrochlorothiazide | Glucuronic acid | 0.301 | 0.142 | 3.41E-02 | 2.12E-01 | 69 | 5 |
| hydrochlorothiazide | 4-Methylcatechol 1-sulfate | 0.272 | 0.139 | 4.97E-02 | 2.47E-01 | 29 | 5 |
| hydrochlorothiazide | Taurocholenate sulfate | 0.268 | 0.109 | 1.39E-02 | 1.51E-01 | 28 | 5 |
| hydrochlorothiazide | Arabinonic acid/Xylonic acid | 0.256 | 0.124 | 3.87E-02 | 2.23E-01 | 75 | 5 |
| hydrochlorothiazide | N(5)-Acetyl-ornithine | 0.249 | 0.085 | 3.30E-03 | 1.18E-01 | 16 | 5 |
| hydrochlorothiazide | 3-Hydroxyoctanoic acid | 0.248 | 0.093 | 7.71E-03 | 1.18E-01 | 45 | 5 |
| hydrochlorothiazide | 3-Methoxytyramine sulfate | 0.242 | 0.113 | 3.19E-02 | 2.12E-01 | 57 | 5 |
| hydrochlorothiazide | Tetradecanedioic acid | 0.242 | 0.098 | 1.35E-02 | 1.51E-01 | 25 | 5 |
| hydrochlorothiazide | 1,5-Anhydrosorbitol | 0.237 | 0.083 | 4.21E-03 | 1.18E-01 | 24 | 5 |
| hydrochlorothiazide | N-Acetylthreonine | 0.230 | 0.094 | 1.41E-02 | 1.51E-01 | 75 | 5 |
| hydrochlorothiazide | Ribonic acid | 0.227 | 0.085 | 7.34E-03 | 1.18E-01 | 42 | 5 |
| hydrochlorothiazide | Orotic acid | 0.224 | 0.094 | 1.75E-02 | 1.67E-01 | 41 | 5 |
| hydrochlorothiazide | CAR 4:0 | 0.221 | 0.081 | 6.33E-03 | 1.18E-01 | 13 | 5 |
| **hydrochlorothiazide** | **Uric acid** | **0.220** | **0.049** | **7.19E-06** | **1.58E-03** | **53** | **5** |
| hydrochlorothiazide | 2-Hydroxyoctanoic acid | 0.220 | 0.099 | 2.62E-02 | 1.92E-01 | 55 | 5 |
| hydrochlorothiazide | 2-Hydroxybutyric acid/2-Hydroxyisobutyric acid | 0.217 | 0.096 | 2.34E-02 | 1.83E-01 | 57 | 5 |
| hydrochlorothiazide | gamma-CEHC | 0.214 | 0.095 | 2.50E-02 | 1.86E-01 | 10 | 5 |
| hydrochlorothiazide | 2-Aminocaprylic acid | 0.213 | 0.102 | 3.64E-02 | 2.18E-01 | 41 | 5 |
| hydrochlorothiazide | CAR 14:1 | 0.210 | 0.078 | 7.26E-03 | 1.18E-01 | 8 | 5 |
| hydrochlorothiazide | N1-Methyl-2-pyridone-5-carboxamide | 0.208 | 0.091 | 2.15E-02 | 1.78E-01 | 31 | 5 |
| hydrochlorothiazide | gamma-Glutamylphenylalanine | 0.206 | 0.093 | 2.74E-02 | 1.97E-01 | 76 | 5 |
| **hydrochlorothiazide** | **Cortisol** | **0.201** | **0.053** | **1.46E-04** | **1.60E-02** | **2** | **5** |
| hydrochlorothiazide | Linoleic acid | 0.199 | 0.100 | 4.66E-02 | 2.44E-01 | 44 | 5 |
| hydrochlorothiazide | 3-Hydroxydodecanoic acid | 0.198 | 0.091 | 2.96E-02 | 2.06E-01 | 18 | 5 |
| hydrochlorothiazide | CAR 10:1 | 0.195 | 0.070 | 5.64E-03 | 1.18E-01 | 10 | 5 |
| hydrochlorothiazide | 2-Ethyl-hydracrylic acid | 0.195 | 0.072 | 6.45E-03 | 1.18E-01 | 45 | 5 |
| hydrochlorothiazide | 3-Hydroxydecanoic acid | 0.194 | 0.070 | 5.38E-03 | 1.18E-01 | 6 | 5 |
| hydrochlorothiazide | N-Acetyltaurine | 0.192 | 0.082 | 2.00E-02 | 1.78E-01 | 56 | 5 |
| hydrochlorothiazide | C-Glycosyltryptophan | 0.191 | 0.095 | 4.50E-02 | 2.44E-01 | 79 | 5 |
| hydrochlorothiazide | Myo-inositol | 0.187 | 0.075 | 1.21E-02 | 1.51E-01 | 32 | 5 |
| hydrochlorothiazide | Urea | 0.186 | 0.066 | 4.83E-03 | 1.18E-01 | 41 | 5 |
| hydrochlorothiazide | CAR 6:0 | 0.186 | 0.064 | 3.72E-03 | 1.18E-01 | 5 | 5 |
| hydrochlorothiazide | Hexadecanedioic acid | 0.184 | 0.089 | 3.83E-02 | 2.23E-01 | 30 | 5 |
| hydrochlorothiazide | CAR 10:0 | 0.182 | 0.086 | 3.36E-02 | 2.12E-01 | 12 | 5 |
| hydrochlorothiazide | Cytidine | 0.175 | 0.077 | 2.25E-02 | 1.82E-01 | 13 | 5 |
| hydrochlorothiazide | Imidazolelactic acid | 0.173 | 0.066 | 8.66E-03 | 1.26E-01 | 42 | 5 |
| **hydrochlorothiazide** | **Fumaric acid** | **0.171** | **0.044** | **8.89E-05** | **1.30E-02** | **3** | **5** |
| hydrochlorothiazide | CAR 12:0 | 0.170 | 0.076 | 2.45E-02 | 1.85E-01 | 10 | 5 |
| hydrochlorothiazide | CAR 8:0 | 0.169 | 0.086 | 4.91E-02 | 2.47E-01 | 16 | 5 |
| hydrochlorothiazide | Retinol | 0.167 | 0.059 | 4.32E-03 | 1.18E-01 | 49 | 5 |
| hydrochlorothiazide | Pseudouridine | 0.158 | 0.075 | 3.48E-02 | 2.12E-01 | 75 | 5 |
| hydrochlorothiazide | 3S-Hydroxyhexanoic acid | 0.157 | 0.056 | 5.17E-03 | 1.18E-01 | 4 | 5 |
| hydrochlorothiazide | CAR 16:1 | 0.151 | 0.076 | 4.78E-02 | 2.44E-01 | 36 | 5 |
| hydrochlorothiazide | Ethylmalonic acid | 0.151 | 0.076 | 4.73E-02 | 2.44E-01 | 30 | 5 |
| hydrochlorothiazide | Cystine | 0.147 | 0.062 | 1.70E-02 | 1.65E-01 | 16 | 5 |
| hydrochlorothiazide | Dimethylglycine | 0.146 | 0.072 | 4.32E-02 | 2.43E-01 | 63 | 5 |
| hydrochlorothiazide | Pyruvic acid | 0.146 | 0.053 | 5.75E-03 | 1.18E-01 | 6 | 5 |
| hydrochlorothiazide | CAR 3:0 | 0.145 | 0.058 | 1.25E-02 | 1.51E-01 | 23 | 5 |
| hydrochlorothiazide | Sulfate | 0.143 | 0.058 | 1.39E-02 | 1.51E-01 | 68 | 5 |
| hydrochlorothiazide | 3-Methoxytyrosine | 0.135 | 0.055 | 1.41E-02 | 1.51E-01 | 14 | 5 |
| hydrochlorothiazide | Lactic acid | 0.134 | 0.044 | 2.20E-03 | 1.18E-01 | 1 | 5 |
| hydrochlorothiazide | Malic acid | 0.131 | 0.045 | 3.70E-03 | 1.18E-01 | 24 | 5 |
| hydrochlorothiazide | N-Acetylalanine | 0.131 | 0.054 | 1.57E-02 | 1.63E-01 | 68 | 5 |
| hydrochlorothiazide | CAR 14:0 | 0.125 | 0.059 | 3.41E-02 | 2.12E-01 | 4 | 5 |
| hydrochlorothiazide | CAR 2:0 | 0.122 | 0.042 | 4.04E-03 | 1.18E-01 | 4 | 5 |
| hydrochlorothiazide | N6-Acetyllysine | 0.118 | 0.044 | 7.80E-03 | 1.18E-01 | 45 | 5 |
| hydrochlorothiazide | CAR 18:2 | 0.116 | 0.055 | 3.48E-02 | 2.12E-01 | 24 | 5 |
| hydrochlorothiazide | N-Acetylhexosamine | 0.113 | 0.056 | 4.29E-02 | 2.43E-01 | 45 | 5 |
| hydrochlorothiazide | cis-Aconitic acid | 0.110 | 0.045 | 1.42E-02 | 1.51E-01 | 29 | 5 |
| hydrochlorothiazide | N-Acetylputrescine | 0.103 | 0.048 | 3.26E-02 | 2.12E-01 | 26 | 5 |
| hydrochlorothiazide | Pyroglutamic acid | 0.097 | 0.036 | 7.32E-03 | 1.18E-01 | 22 | 5 |
| hydrochlorothiazide | Sphingosine 1-phosphate | 0.095 | 0.047 | 4.44E-02 | 2.44E-01 | 24 | 5 |
| hydrochlorothiazide | Taurine | 0.087 | 0.036 | 1.60E-02 | 1.63E-01 | 6 | 5 |
| hydrochlorothiazide | Thyroxine | 0.082 | 0.034 | 1.68E-02 | 1.65E-01 | 5 | 5 |
| hydrochlorothiazide | 1-Methyladenosine | 0.076 | 0.038 | 4.53E-02 | 2.44E-01 | 53 | 5 |
| hydrochlorothiazide | Carnitine | 0.074 | 0.027 | 6.03E-03 | 1.18E-01 | 4 | 5 |

**Supplementary Table 6. Differential metabolites found by meta-analysis to be associated with naproxen intake.** Bold font represents metabolites remaining significantly associated (p<0.05) after FDR correction. MS instrument was not included as a moderator in meta-analysis as there were less than 3 QTOF studies in each case.

| drug | metabolite | LogFC | SE | p_val | p.adj | I2 | number of studies |
| --- | --- | --- | --- | --- | --- | --- | --- |
| **naproxen** | **Glucuronic acid** | **0.954** | **0.185** | **2.47E-07** | **1.02E-04** | **81** | **5** |
| naproxen | 4-Pyridoxic acid | 0.423 | 0.132 | 1.39E-03 | 5.57E-02 | 15 | 5 |
| naproxen | Niacinamide | 0.258 | 0.126 | 4.09E-02 | 3.67E-01 | 69 | 5 |
| naproxen | alpha-N-Phenylacetylglutamine | 0.247 | 0.117 | 3.47E-02 | 3.48E-01 | 28 | 5 |
| naproxen | Urea | 0.243 | 0.086 | 4.66E-03 | 1.13E-01 | 56 | 5 |
| naproxen | CAR 26:1 | -0.104 | 0.052 | 4.34E-02 | 3.67E-01 | 7 | 5 |
| naproxen | Uric acid | -0.133 | 0.065 | 4.28E-02 | 3.67E-01 | 60 | 5 |
| naproxen | gamma-Glutamyllysine | -0.144 | 0.059 | 1.46E-02 | 2.15E-01 | 29 | 5 |
| naproxen | Imidazolelactic acid | -0.144 | 0.070 | 4.11E-02 | 3.67E-01 | 36 | 5 |
| naproxen | Juniperic acid | -0.147 | 0.067 | 2.74E-02 | 3.13E-01 | 18 | 5 |
| naproxen | N-6-Trimethyllysine | -0.150 | 0.064 | 1.95E-02 | 2.65E-01 | 14 | 5 |
| naproxen | Ketoleucine | -0.160 | 0.081 | 4.75E-02 | 3.76E-01 | 54 | 5 |
| naproxen | CAR 24:1 | -0.166 | 0.079 | 3.63E-02 | 3.56E-01 | 29 | 5 |
| naproxen | Isovaleric acid | -0.214 | 0.088 | 1.51E-02 | 2.15E-01 | 32 | 5 |
| **naproxen** | **Kynurenine** | **-0.241** | **0.067** | **3.58E-04** | **2.11E-02** | **56** | **5** |
| naproxen | Methionine sulfone | -0.243 | 0.093 | 8.87E-03 | 1.59E-01 | 51 | 5 |
| naproxen | Dihomo-gamma-linolenic acid | -0.246 | 0.115 | 3.19E-02 | 3.31E-01 | 47 | 5 |
| naproxen | Capric acid | -0.249 | 0.099 | 1.17E-02 | 1.93E-01 | 26 | 5 |
| naproxen | Caprylic acid | -0.255 | 0.088 | 3.64E-03 | 9.38E-02 | 16 | 5 |
| naproxen | CAR DC6:0 | -0.288 | 0.127 | 2.37E-02 | 2.89E-01 | 45 | 5 |
| naproxen | Myristoleic acid | -0.299 | 0.146 | 4.09E-02 | 3.67E-01 | 40 | 5 |
| naproxen | 3-Methylhistidine | -0.327 | 0.146 | 2.51E-02 | 2.95E-01 | 31 | 5 |
| **naproxen** | **Tryptophan** | **-0.328** | **0.067** | **1.12E-06** | **2.32E-04** | **72** | **5** |
| **naproxen** | **2-Hydroxyoctanoic acid** | **-0.333** | **0.087** | **1.18E-04** | **9.73E-03** | **22** | **5** |
| naproxen | 3-Hydroxydecanoic acid | -0.333 | 0.148 | 2.38E-02 | 2.89E-01 | 66 | 5 |
| naproxen | Bilirubin | -0.344 | 0.167 | 3.92E-02 | 3.67E-01 | 72 | 5 |
| naproxen | 11Z,14Z-Eicosadienoic acid | -0.346 | 0.173 | 4.51E-02 | 3.71E-01 | 70 | 5 |
| **naproxen** | **Phenyllactic acid** | **-0.352** | **0.094** | **1.87E-04** | **1.29E-02** | **56** | **5** |
| naproxen | 2-Hydroxycapric acid | -0.357 | 0.134 | 7.95E-03 | 1.52E-01 | 63 | 5 |
| naproxen | 5alpha-pregnan-3beta_20alpha-diol monosulfate (2) | -0.359 | 0.164 | 2.88E-02 | 3.20E-01 | 34 | 5 |
| naproxen | N-Acetyl-tryptophan | -0.369 | 0.114 | 1.23E-03 | 5.57E-02 | 56 | 5 |
| naproxen | Dodecanedioic acid | -0.373 | 0.136 | 6.30E-03 | 1.30E-01 | 49 | 5 |
| naproxen | EPA | -0.379 | 0.120 | 1.60E-03 | 5.57E-02 | 24 | 5 |
| naproxen | S-Allylcysteine | -0.387 | 0.192 | 4.36E-02 | 3.67E-01 | 13 | 5 |
| naproxen | 21-Hydroxypregnenolone | -0.399 | 0.160 | 1.28E-02 | 1.97E-01 | 50 | 5 |
| naproxen | DPA | -0.412 | 0.156 | 8.14E-03 | 1.52E-01 | 55 | 5 |
| naproxen | Phenyl hydrogen sulfate | -0.414 | 0.139 | 2.96E-03 | 8.72E-02 | 30 | 5 |
| naproxen | Pregnenolone sulfate | -0.416 | 0.132 | 1.62E-03 | 5.57E-02 | 32 | 5 |
| naproxen | Eicosanedioic acid | -0.426 | 0.197 | 3.04E-02 | 3.30E-01 | 81 | 5 |
| **naproxen** | **10-Hendecenoic acid** | **-0.432** | **0.103** | **2.71E-05** | **2.79E-03** | **35** | **5** |
| naproxen | 5-Dodecenoic acid | -0.465 | 0.217 | 3.21E-02 | 3.31E-01 | 74 | 5 |
| naproxen | 4-Hydroxystyrene | -0.467 | 0.202 | 2.10E-02 | 2.70E-01 | 31 | 5 |
| naproxen | 4-Androsten-3beta,17beta-diol disulfate | -0.484 | 0.174 | 5.42E-03 | 1.24E-01 | 30 | 5 |
| naproxen | androstenediol (3beta_17beta) monosulfate (1) | -0.515 | 0.188 | 6.26E-03 | 1.30E-01 | 46 | 5 |
| naproxen | Octadecanedioic acid | -0.523 | 0.204 | 1.04E-02 | 1.78E-01 | 69 | 5 |
| naproxen | Stearidonic acid | -0.525 | 0.211 | 1.29E-02 | 1.97E-01 | 67 | 5 |
| naproxen | Epiandrosterone | -0.533 | 0.229 | 2.00E-02 | 2.65E-01 | 52 | 5 |
| naproxen | 3-Indolepropionic acid | -0.554 | 0.189 | 3.30E-03 | 9.07E-02 | 44 | 5 |
| naproxen | Dehydroepiandrosterone sulfate | -0.573 | 0.176 | 1.15E-03 | 5.57E-02 | 47 | 5 |
| **naproxen** | **Indolelactic acid** | **-0.618** | **0.131** | **2.57E-06** | **3.53E-04** | **76** | **5** |
| naproxen | Tetradecanedioic acid | -0.669 | 0.336 | 4.66E-02 | 3.76E-01 | 92 | 5 |
| naproxen | 4-Allylphenol sulfate | -0.673 | 0.219 | 2.14E-03 | 6.78E-02 | 50 | 5 |

**Supplementary Table 7. Differential metabolites found by meta-analysis to be associated with metformin intake.** Bold font represents metabolites remaining significantly associated (p<0.05) after FDR correction. MS instrument was not included as a moderator in meta-analysis as there were less than 3 QTOF studies in each case.

| drug | metabolite | LogFC | SE | p_val | p.adj | I2 | number of studies |
| --- | --- | --- | --- | --- | --- | --- | --- |
| metformin | Trimethylamine N-oxide | 0.311 | 0.150 | 3.74E-02 | 1.95E-01 | 39 | 5 |
| **metformin** | **Glutamic acid** | **0.258** | **0.071** | **2.84E-04** | **6.67E-03** | **29** | **5** |
| metformin | Pantothenic acid | 0.206 | 0.095 | 3.00E-02 | 1.95E-01 | 31 | 5 |
| **metformin** | **Proline** | **0.171** | **0.044** | **1.14E-04** | **5.34E-03** | **13** | **5** |
| metformin | Threonine | -0.089 | 0.042 | 3.64E-02 | 1.95E-01 | 10 | 5 |
| metformin | Tryptophan | -0.096 | 0.036 | 8.00E-03 | 6.27E-02 | 13 | 5 |
| **metformin** | **Arginine** | **-0.212** | **0.075** | **4.84E-03** | **4.55E-02** | **70** | **5** |
| **metformin** | **Citrulline** | **-0.442** | **0.144** | **2.12E-03** | **2.50E-02** | **90** | **5** |
| **metformin** | **N-6-Trimethyllysine** | **-0.532** | **0.167** | **1.42E-03** | **2.23E-02** | **86** | **5** |

**Supplementary Table 8. Differential metabolites found by meta-analysis to be associated with ibuprofen intake.** Bold font represents metabolites remaining significantly associated (p<0.05) after FDR correction. MS instrument was not included as a moderator in meta-analysis as there were less than 3 QTOF studies in each case.

| drug | metabolite | LogFC | SE | p_val | p.adj | I2 | number of studies |
| --- | --- | --- | --- | --- | --- | --- | --- |
| **ibuprofen** | **Glucuronic acid** | **0.925** | **0.167** | **3.04E-08** | **1.33E-05** | **72** | **5** |
| ibuprofen | 5-Acetylamino-6-amino-3-methyluracil | 0.695 | 0.273 | 1.10E-02 | 6.87E-01 | 24 | 5 |
| ibuprofen | Sucrose | 0.598 | 0.187 | 1.34E-03 | 1.96E-01 | 14 | 5 |
| ibuprofen | 1-Methyluric acid | 0.574 | 0.251 | 2.22E-02 | 8.65E-01 | 38 | 5 |
| ibuprofen | Theophylline | 0.572 | 0.248 | 2.09E-02 | 8.65E-01 | 26 | 5 |
| ibuprofen | N4-Acetylcytidine | 0.451 | 0.184 | 1.45E-02 | 7.93E-01 | 59 | 5 |
| ibuprofen | 1,3-Dimethyluric acid | 0.443 | 0.220 | 4.44E-02 | 8.65E-01 | 43 | 5 |
| ibuprofen | gamma-CEHC glucuronide | 0.406 | 0.148 | 6.04E-03 | 4.96E-01 | 22 | 5 |
| ibuprofen | Indole-3-methyl acetate | 0.330 | 0.140 | 1.88E-02 | 8.65E-01 | 8 | 5 |
| ibuprofen | Lauric acid | 0.255 | 0.124 | 3.95E-02 | 8.65E-01 | 40 | 5 |
| ibuprofen | Indoleacetic acid | 0.183 | 0.091 | 4.43E-02 | 8.65E-01 | 2 | 5 |
| ibuprofen | N-Acetylphenylalanine | 0.165 | 0.077 | 3.19E-02 | 8.65E-01 | 20 | 5 |
| ibuprofen | Asparagine | -0.065 | 0.030 | 3.04E-02 | 8.65E-01 | 1 | 5 |
| ibuprofen | Guanidoacetic acid | -0.118 | 0.058 | 4.21E-02 | 8.65E-01 | 7 | 5 |
| ibuprofen | 3-Methyl-2-oxovaleric acid | -0.138 | 0.051 | 6.81E-03 | 4.96E-01 | 13 | 5 |
| **ibuprofen** | **Tryptophan** | **-0.141** | **0.038** | **1.85E-04** | **4.04E-02** | **9** | **5** |
| ibuprofen | Ketoleucine | -0.160 | 0.051 | 1.80E-03 | 1.97E-01 | 7 | 5 |
| ibuprofen | gamma-Glutamylmethionine | -0.190 | 0.088 | 3.14E-02 | 8.65E-01 | 47 | 5 |
| ibuprofen | gamma-Glutamyl-2-aminobutanoate | -0.299 | 0.142 | 3.45E-02 | 8.65E-01 | 27 | 5 |
| ibuprofen | Glycohyocholic acid | -0.308 | 0.152 | 4.28E-02 | 8.65E-01 | 9 | 5 |
| ibuprofen | Sulfoglycolithocholic acid | -0.317 | 0.150 | 3.42E-02 | 8.65E-01 | 1 | 5 |

**Supplementary Table 9. Differential metabolites found by meta-analysis to be associated with ranitidine intake.** Bold font represents metabolites remaining significantly associated (p<0.05) after FDR correction. MS instrument was not included as a moderator in meta-analysis as there were less than 3 QTOF studies in each case.

| drug | metabolite | LogFC | SE | p_val | p.adj | I2 | number of studies |
| --- | --- | --- | --- | --- | --- | --- | --- |
| **ranitidine** | **Sucrose** | **1.302** | **0.297** | **1.18E-05** | **7.35E-03** | **29** | **6** |
| ranitidine | Salicyluric acid | 0.996 | 0.485 | 3.99E-02 | 7.76E-01 | 40 | 6 |
| ranitidine | Glucuronic acid | 0.633 | 0.218 | 3.65E-03 | 3.11E-01 | 65 | 6 |
| ranitidine | N-Acetyl-3-methylhistidine | 0.546 | 0.184 | 3.02E-03 | 3.11E-01 | 8 | 5 |
| ranitidine | Ectoine | 0.511 | 0.190 | 7.02E-03 | 3.94E-01 | 4 | 6 |
| ranitidine | 4-Hydroxyhippuric acid | 0.475 | 0.167 | 4.50E-03 | 3.11E-01 | 1 | 6 |
| ranitidine | Phenyl acetate | 0.473 | 0.216 | 2.87E-02 | 6.72E-01 | 29 | 5 |
| ranitidine | 4-Hydroxyphenyl acetate | 0.470 | 0.211 | 2.58E-02 | 6.68E-01 | 41 | 5 |
| ranitidine | Lanthionine | 0.455 | 0.215 | 3.43E-02 | 7.62E-01 | 38 | 5 |
| ranitidine | N-Acetylcitrulline | 0.439 | 0.161 | 6.47E-03 | 3.94E-01 | 24 | 5 |
| ranitidine | N-Acetylphenylalanine | 0.322 | 0.101 | 1.51E-03 | 2.81E-01 | 17 | 6 |
| ranitidine | N-Acetylarginine | 0.309 | 0.099 | 1.81E-03 | 2.81E-01 | 13 | 6 |
| ranitidine | 5'-Methylthioadenosine | 0.276 | 0.140 | 4.93E-02 | 7.87E-01 | 62 | 6 |
| ranitidine | 3-Hydroxymethylglutaric acid | 0.274 | 0.132 | 3.75E-02 | 7.76E-01 | 47 | 6 |
| ranitidine | 6-oxopiperidine-2-carboxylic acid | 0.267 | 0.130 | 3.93E-02 | 7.76E-01 | 23 | 6 |
| ranitidine | Pro-hydroxyPro | 0.258 | 0.116 | 2.57E-02 | 6.68E-01 | 20 | 6 |
| ranitidine | N-Acetylleucine | 0.239 | 0.099 | 1.60E-02 | 5.17E-01 | 34 | 6 |
| ranitidine | CAR DC4:0 | 0.214 | 0.109 | 4.94E-02 | 7.87E-01 | 36 | 6 |
| ranitidine | N-Acetylhexosamine | 0.176 | 0.074 | 1.73E-02 | 5.17E-01 | 10 | 6 |
| ranitidine | Creatinine | 0.134 | 0.047 | 4.42E-03 | 3.11E-01 | 15 | 6 |
| ranitidine | PC 16:0/18:2 | -0.088 | 0.037 | 1.83E-02 | 5.17E-01 | 33 | 6 |
| ranitidine | Pentadecylic acid | -0.212 | 0.097 | 2.82E-02 | 6.72E-01 | 30 | 6 |
| ranitidine | Adrenic acid | -0.245 | 0.124 | 4.72E-02 | 7.87E-01 | 6 | 6 |
| ranitidine | Margaric acid | -0.271 | 0.109 | 1.33E-02 | 4.87E-01 | 9 | 6 |
| ranitidine | gamma-Glutamyl-2-aminobutanoate | -0.293 | 0.123 | 1.73E-02 | 5.17E-01 | 5 | 5 |
| ranitidine | CAR 17:0 | -0.315 | 0.124 | 1.08E-02 | 4.48E-01 | 22 | 5 |
| ranitidine | Dodecanedioic acid | -0.388 | 0.152 | 1.04E-02 | 4.48E-01 | 19 | 6 |
| ranitidine | 5alpha-pregnan-3beta_20alpha-diol monosulfate (2) | -0.427 | 0.211 | 4.34E-02 | 7.87E-01 | 16 | 6 |
| ranitidine | 21-Hydroxypregnenolone | -0.551 | 0.279 | 4.82E-02 | 7.87E-01 | 66 | 6 |
| ranitidine | 5alpha-androstan-3alpha_17beta-diol monosulfate (1) | -0.573 | 0.242 | 1.79E-02 | 5.17E-01 | 23 | 5 |
| ranitidine | Taurolithocholic acid sulfate | -0.630 | 0.289 | 2.92E-02 | 6.72E-01 | 34 | 5 |
| ranitidine | 5alpha-Pregnan-3beta,20alpha-diol disulfate | -0.658 | 0.261 | 1.15E-02 | 4.49E-01 | 39 | 6 |
| ranitidine | androstenediol (3alpha_ 17alpha) monosulfate (3) | -0.660 | 0.247 | 7.61E-03 | 3.94E-01 | 31 | 5 |
| ranitidine | 5alpha-Androstane-3beta,17beta-diol disulfate | -0.687 | 0.334 | 3.98E-02 | 7.76E-01 | 41 | 6 |
| ranitidine | Hydrocinnamic acid | -0.786 | 0.271 | 3.79E-03 | 3.11E-01 | 16 | 5 |
| ranitidine | Cinnamoylglycine | -1.007 | 0.291 | 5.41E-04 | 1.68E-01 | 5 | 5 |
| ranitidine | thymol sulfate | -1.243 | 0.476 | 9.00E-03 | 4.31E-01 | 39 | 5 |

**Supplementary Table 10. Differential metabolites found by meta-analysis to be associated with gabapentin intake.** Bold font represents metabolites remaining significantly associated (p<0.05) after FDR correction. MS instrument was not included as a moderator in meta-analysis as there were less than 3 QTOF studies in each case.

| drug | metabolite | LogFC | SE | p_val | p.adj | I2 | number of studies |
| --- | --- | --- | --- | --- | --- | --- | --- |
| gabapentin | 4-Pyridoxic acid | 0.900 | 0.347 | 9.50E-03 | 1.20E-01 | 65 | 5 |
| gabapentin | N1-Methyl-2-pyridone-5-carboxamide | 0.447 | 0.191 | 1.92E-02 | 1.35E-01 | 46 | 5 |
| gabapentin | Pantothenic acid | 0.396 | 0.162 | 1.46E-02 | 1.20E-01 | 56 | 5 |
| **gabapentin** | **N4-Acetylcytidine** | **0.377** | **0.108** | **4.61E-04** | **2.26E-02** | **20** | **5** |
| gabapentin | 1-Methyladenosine | 0.150 | 0.061 | 1.39E-02 | 1.20E-01 | 36 | 5 |
| gabapentin | Tryptophan | -0.137 | 0.046 | 3.23E-03 | 7.90E-02 | 4 | 5 |
| gabapentin | 3-Dehydroxycarnitine | -0.141 | 0.066 | 3.11E-02 | 1.70E-01 | 25 | 5 |
| gabapentin | sn-Glycero-3-phosphocholine | -0.266 | 0.120 | 2.71E-02 | 1.66E-01 | 55 | 5 |
| gabapentin | Xanthine | -0.338 | 0.136 | 1.31E-02 | 1.20E-01 | 35 | 5 |

**Supplementary Table 11. Differential metabolites found by meta-analysis to be associated with diphenhydramine intake.** Bold font represents metabolites remaining significantly associated (p<0.05) after FDR correction. MS instrument was not included as a moderator in meta-analysis as there were less than 3 QTOF studies in each case.

| drug | metabolite | LogFC | SE | p_val | p.adj | I2 | number of studies |
| --- | --- | --- | --- | --- | --- | --- | --- |
| diphenhydramine | LPE 16:0/0:0 | 0.178 | 0.067 | 7.43E-03 | 4.09E-01 | 7 | 5 |
| diphenhydramine | Methylimidazoleacetic acid | 0.173 | 0.079 | 2.79E-02 | 7.33E-01 | 1 | 5 |
| diphenhydramine | Glutamic acid | 0.168 | 0.082 | 4.00E-02 | 7.33E-01 | 19 | 5 |
| diphenhydramine | 1-Methyladenosine | 0.132 | 0.041 | 1.33E-03 | 1.47E-01 | 13 | 5 |
| diphenhydramine | LPC 16:0/0:0 | 0.102 | 0.043 | 1.67E-02 | 6.12E-01 | 11 | 5 |
| diphenhydramine | Indoleacetic acid | -0.210 | 0.101 | 3.66E-02 | 7.33E-01 | 1 | 5 |
